# Genomic and phenotypic characterization of *Pseudomonas aeruginosa* isolates from two Mexican cystic fibrosis attention centers

**DOI:** 10.1128/spectrum.01100-24

**Published:** 2024-10-23

**Authors:** Luis Ángel Núñez-García, José Manuel Feliciano-Guzmán, Christian Daniel Mireles-Davalos, José Raúl López-Sántiz, Jesús Elías Ovando-Fonseca, Eduardo Becerril-Vargas, María Elena Jiménez-Martínez, Nadia Rodríguez-Medina, Ulises Garza-Ramos, Carlos Córdova-Fletes, Elvira Garza-González

**Affiliations:** 1 Departamento de Bioquímica y Medicina Molecular, Facultad de Medicina, Universidad Autónoma de Nuevo León, Monterrey, Nuevo León, Mexico; 2 Hospital de Especialidades Pediátricas, Tuxtla Gutiérrez, Chiapas, Mexico; 3 Instituto Nacional de Enfermedades Respiratorias Ismael Cosío Villegas, Ciudad de Mexico, Mexico; 4 Instituto Nacional de Salud Pública, Laboratorio de Resistencia Bacteriana, Cuernavaca, Morelos, Mexico; University of Manitoba, Winnipeg, Manitoba, Canada

**Keywords:** Cystic fibrosis, *Pseudomonas aeruginosa*, Bioinformatics, Genomic, Phenotypic testing, Adaptation, Mobilome, Elastase

## Abstract

Thirty-nine clinical isolates of *Pseudomonas aeruginosa* collected from 11 cystic fibrosis (CF) patients at two CF attention centers over 10 years were subjected to whole genome sequencing (WGS). Phenotypic tests (i.e., elastase, motility, biofilm, growth rate, and antibiotic susceptibility) were performed to correlate results. A single strain of *P. aeruginosa* was found to persist over time in longitudinal isolates. No transmission between patients or centers was observed. A tendency to lack genes related to pyoverdine, flagellum, pili, and O-antigen was observed, whereas those related to biofilm, phenazine, and pyochelin were conserved among isolates. In a patient with a 10-year follow-up, a single strain of *P. aeruginosa* persisted and showed a gradual decrease in elastase activity and growth rate, demonstrating an adaptive phenotype.

**IMPORTANCE:**

This study investigates the genomic and phenotypic characteristics of *Pseudomonas aeruginosa* isolates from Mexican cystic fibrosis (CF) patients, an underrepresented group in CF research. To our knowledge, it is the first to use whole genome sequencing (WGS) to study longitudinally collected *P. aeruginosa* isolates from this population, evaluating both genomic features and clonal relationships. Remarkably, the study includes samples from one patient over 10 years, offering an extended observation time compared to existing literature. Unlike similar studies, which often lack phenotypic testing, this research incorporates various virulence-related phenotypic assays, enhancing our understanding of gene-to-phenotype correlations. Two potential mechanisms for the loss of elastolytic activity were identified. Furthermore, we conduct an in-depth mobilome analysis, an area that remains largely unexplored in CF contexts. Whole genome sequencing data are publicly available through the NCBI SRA database, facilitating further re-analysis for studies on *P. aeruginosa* in CF, as well as epidemiological and population structure research.

## INTRODUCTION

Cystic fibrosis (CF) is one of the most common genetic diseases, affecting approximately 150,000 individuals worldwide (1). Due to advances in therapy, the life expectancy of patients has increased from 11 years in 1980 to 50 years in some countries today (2). The resulting increase in the adult people with CF population has introduced new therapeutic challenges, as complications such as CF-related diabetes, kidney disease, non-malignant gastrointestinal changes, metabolic bone disease, and decrease in lung function have increased in prevalence as people with CF age (3). Lung disease is the most common manifestation among older people with CF and has become the leading cause of mortality among this population (4–7).

Lung disease may involve a broad spectrum of microorganisms that colonize and infect the CF patient’s airway. However, *Pseudomonas aeruginosa* is the most common bacterial species found in older people with CF (8). The presence of diverse virulence factors and resistance potential to a broad spectrum of antibiotics facilitate the colonization and adaptation of *P. aeruginosa* to the airways of people with CF (9–13).

*P. aeruginosa* exhibits a diverse array of virulence factors that facilitate early colonization and establishment in the CF-patient lung; however, adapted strains tend to display a loss or diminishment of traits such as type-IV pili and flagellum-dependent motility, O-antigen biosynthesis (11), growth rate (14), and elastolytic activity (15). On the other hand, transition to mucoid phenotypes due to an overproduction of alginate and an aggregative, biofilm-associated lifestyle are commonly observed in this context (9, 16). Surface components (pili, flagella, and O-antigen) mediate immune system recognition, and thus it is thought that loss of these components plays a role in decreasing immune recognition (11), while the diminishment of growth rate (17), transition to mucoid phenotypes, and biofilm lifestyle provide an enhanced antibiotic resistance (18–20).

Longitudinal studies have revealed the existence of genomic, transcriptomic, proteomic, and phenotypic profiles shared by non-related *P. aeruginosa* strains isolated from CF airways (16).

Although whole genome sequencing (WGS) studies have identified genes prone to accumulate mutations and have allowed them to elucidate mutation rates and adaptation mechanisms, few of these studies have enriched genomic findings with phenotypic evaluations, which represents a challenge in correlating genomic and phenotypic characteristics. For example, the loss or mutation of flagellum-related genes is frequent among *P. aeruginosa* strains isolated from CF airways, but some groups report a loss or absence of flagellum-dependent motility (21–23), while others observe a high frequency and longitudinal maintenance of this type of motility (24, 25).

Additionally, *P. aeruginosa* isolated from Mexican CF patients remains underexplored at the genomic level, representing a notable knowledge gap in this population. Therefore, our study aims to correlate the adaptive changes and relatedness of longitudinally collected *P. aeruginosa* isolates from CF patients at two CF care centers in Mexico and complement these findings with genomics approaches, identifying the persistence of *P. aeruginosa* isolates in patients with CF, as well as evaluating patient-to-patient transmission.

## MATERIALS AND METHODS

### Study sites and clinical isolates

Clinical respiratory isolates of *P. aeruginosa* from pediatric CF patients at two Mexican CF attention centers—the Hospital de Especialidades Pediátricas (HEP) (https://testwebqa.cndh.org.mx/palabras-clave/6244/hospital-de-especialidades-pediatricas) and the Instituto Nacional de Enfermedades Respiratorias (INER) (https://www.gob.mx/salud/iner)—were included in the sample. Isolates were collected from patients and stored at −80°C in nutritive broth containing 15% glycerol.

The sample consisted of a total of 39 isolates, of which 32 were longitudinally collected from four different patients (patients 1–4), with 19, 6, 5, and 2 isolates collected, respectively, and seven of which corresponded to single-patient isolates (patients 5–11).

### Whole genome sequencing

Cryo-preserved clinical isolates were thawed and cultured on MacConkey agar (Becton-Dickinson, Massachusetts, USA). Genomic DNA was extracted using the phenol-chloroform method (26). The quality of the DNA was assessed using UV spectrophotometry. A concentration greater than 10 ng/µL, a total yield greater than 200 ng, and a 260/280 ratio between 1.8 and 2.0 were deemed appropriate for further analysis. WGS was performed by an external service (Novogene Corporation Inc, USA) using an Illumina NovaSeq 6000 instrument (Illumina, California, USA).

### Genome assembly and bioinformatic analysis

Genome assembly was completed using the Illumina DRAGEN secondary analysis pipeline with raw sequencing data. The *de novo* assemblies generated were used for virulome, resistome, and multilocus sequence typing (MLST) analysis, and wgMLST-generated phylogenetic trees were analyzed using the automated software Epi-Seq (BioMérieux, France).

For manual bioinformatic analysis, the TrimGalore v0.4.4_dev (https://github.com/FelixKrueger/TrimGalore; accessed on 12 July 2023) software was used to remove adapter content of raw reads, and genomes were re-assembled using Unicycler v0.4.9b (27). Assembly quality was assessed using CheckM v1.2.2 (28) and QUAST v5.2.0 (29) to evaluate genome completeness and contamination, as well as standard quality metrics. Snippy v4.6.0 (30) was used for variant calling, in which raw reads were mapped against the *P. aeruginosa* PAO1 annotated reference genome (GenBank accession GCF_000006765.1; obtained from the *Pseudomonas* genome database: pseudomonas.com). The resulting core and complete genome variant alignment files were used for maximum-likelihood phylogenetic reconstruction using RAxML v8.2.12 (31), as follows: “raxmlHPC-PTHREADS-AVX2 -m GTRCAT -p 1234.” To analyze high-impact variants, synonymous and missense mutations were filtered out from the resulting snps.tab files generated by snippy. Gene ontology (GO) terms related to general regulation, motility, biofilm and secretion systems were obtained from the *Pseudomonas* genome database to extract gene sequences related to these GO terms from the filtered snps.tab files using the corresponding locus tags. Furthermore, a BLAST bit score ratio matrix for each GO term was generated for longitudinal samples to identify mutational changes over time using the LS-BSR software (32) . Multiple sequence alignment (MSA) was performed to analyze genes with longitudinal changes using MAFFT v7.525 (33).

Additionally, the first isolate of longitudinal samples was annotated using Prokka v1.14.5 (34) and was used as a reference for subsequent sample variant calling using the previously described snippy workflow to analyze mutation rates.

Pangenome analysis was completed using the ANVI’O v8 (35) pangenome analysis workflow. Briefly, contig databases were generated (anvi-gen-contigs-database) and annotated (anvi-run-hmms, anvi-run-ncbi-cogs, anvi-scan-trnas, and anvi-run-scg-taxonomy), and the pangenome was calculated using the anvi-pan-genome program (flags –minbit 0.5–mcl-inflation 10 and–use-ncbi-blast). Accessory and core genome size were determined using anvi-script-compute-bayesian-pan-core, which uses mOTUpan (36).

For mobilome analysis, bacteriophages and prophages were identified using PHASTEST (https://phastest.ca/) (37), Mobile Element Finder v1.1.2 (38) for integrative conjugative and mobilizable elements (ICEs and IMEs, respectively), insertion sequences (IS), and transposons (Tn). MOB-suite (39) was used for plasmid prediction, and the resulting putative plasmids were introduced in the Basic Local Alignment Search Tool (BLAST) (https://blast.ncbi.nlm.nih.gov/Blast.cgi) web service and discarded if they produced significant alignments with chromosome sequences. The identified mobilome sequences were annotated using the previously described anvi’o workflow, and COG20 categories were extracted for each isolate’s mobile elements. Because *P. aeruginosa* phage-like bacteriocins (R- and F-type pyocins) share homology with bacteriophages, sequences from the PAO1 reference strain for R- and F-type pyocins were extracted (genomic coordinates 675861-687946 and 689236-703058, respectively) and aligned against the PHASTEST putative phage sequences using BLAST (40).

### Antibiotic susceptibility testing

Identification and susceptibility tests of clinical isolates were performed using the Vitek-2 instrument (BioMérieux, France).

### Motility and morphology assays

Twitching, swarming, and swimming motility were evaluated using plate-based assays, according to the protocols described by Filoux and colleagues (41).

### Elastolytic activity

Comparative semi-quantitative elastase activity was assessed in longitudinal isolates. Briefly, bacterial suspensions corresponding to a 0.5-MacFarland tube were prepared in sterile saline, and 2.33 µL of these suspensions was transferred to 4 mL LB-Lennox broth and was cultured for 24 hours at 37°C. Cultures were centrifuged at 3,000 × *g* for 10 minutes at 4°C. Supernatants were 1:1 diluted with 1× reaction buffer, and elastase activity was evaluated using the EnzCheck Elastase assay kit (Invitrogen, USA), according to the manufacturer’s instructions.

Sterile LB broth and 0.25 U/mL porcine pancreatic elastase standard were used as negative and positive controls, respectively. All measurements were performed in triplicate on two separate days. Samples corresponding to the same patient were tested in the same run to ensure reproducibility.

### Specific growth rate (µ)

The specific growth rate was evaluated by transferring 200 mL of bacterial suspension equivalent to a 0.25-MacFarland standard in Mueller-Hinton broth to sterile, flat-bottomed 96-well microplates (Sarstedt, Germany). The lids were sealed with parafilm paper, and the plates were incubated for 24 hours at 37°C in a Cytation 1 (BioTek Instruments, USA) microplate reader. The optical density (OD600) of each sample was monitored every 10 minutes, with 10 seconds of shaking prior to each measurement. The exponential phase of cultures was identified and used to calculate the specific growth rate (µ), which corresponds to the slope of the curve when plotting the ln(OD600) against time. Experiments were performed on separate days, in which four technical replicates were included. Data were determined to follow a normal distribution by the Shapiro-Wilk test and were further compared against the first isolate using Dunnett’s test.

### Biofilm production

Biofilm production capacity was evaluated using the method described by Stepanović et al. (42), with modifications. Briefly, overnight, LB Lennox cultures were used to prepare bacterial suspensions equivalent to a 0.5-MacFarland standard. Then, 20 µL of these suspensions was transferred to sterile, flat-bottomed 96-well microplates containing 180 µL of soy-trypticase broth with 1% glucose and was incubated for 24 hours. Afterward, planktonic growth was decanted, and biofilms were washed thrice with sterile phosphate buffer. Absolute methanol (CTR Scientific, Mexico) was added, and the plates were incubated for 20 minutes. The methanol was removed, and the plates were incubated for 1 hour at 60°C. Biofilm was stained with 0.1% crystal violet for 15 minutes. Then, the colorant was removed, and stained biofilms were washed five times with sterile distilled water. Finally, 30% acetic acid (CTR Scientific, Mexico) was added and incubated for 30 minutes, and 570-nm absorbance (A) was measured in a Cytation one microplate. The A of the blank (standard deviation adjusted) was used to classify the strains as non-biofilm producers (A < blank), weak producers (blank < A < 2*blank), moderate producers (2*blank < A < 4*blank), and strong producers (4*blank < A) (42).

## RESULTS

### Clinical isolates

Longitudinal isolates from patient 1 included 19 isolates collected over 10 years (2013–2023), whereas isolates from patients 2 (six isolates) and 3 (five isolates) were collected over 3 years (2014–2017). Isolates from patient 4 included two isolates collected in 2019 and 2020. The date of collection of clinical isolates is shown in Table 1. Patients 1, 2, 3, 6, and 7 were treated at HEP, and patients 4, 8, 9, 10, and 11 were treated at INER.

**TABLE 1:** Collection date, MLST, and antibiotic susceptibility testing of strains^*a*^

Patient	Strain	Isolation date	MLST	AMK	FEP	CAZ	CZA	CIP	CT	IMP	MEM	TZP
1	A697	01/04/2013	829	S	S	S	S	S	S	S	S	S
1	A703	27/02/2014	829	S	I	S	S	S	S	R	R	I
1	A705	12/12/2014	82	S	I	S	S	S	S	S	S	S
1	A701	16/04/2015	829	S	S	S	S	S	S	S	S	S
1	A710	10/12/2015	829	S	S	S	S	S	S	S	S	S
1	A734	19/04/2016	829	S	S	S	S	I	S	S	S	I
1	A748	02/09/2016	829	S	S	S	S	R	S	S	S	I
1	A768	24/04/2017	829	S	S	S	S	R	S	S	S	S
1	A780	22/11/2017	829	S	S	S	S	S	S	S	S	S
1	A778	02/03/2018	934	S	S	S	S	S	S	S	S	S
1	AG774	12/10/2018	829	S	S	S	ND	R	ND	S	S	S
1	AP774	12/10/2018	829	S	I	S	ND	R	ND	S	I	S
1	A771	11/11/2020	1800	S	S	S	S	S	S	S	S	S
1	A2152	02/07/2021	829	R	S	S	S	I	S	S	S	S
1	A2155	03/11/2021	829	R	S	S	S	S	S	S	S	ND
1	A2158	09/02/2022	829	R	S	S	S	I	S	S	S	S
1	AG2160	28/06/2022	829	S	S	S	ND	R	ND	S	S	S
1	AP2160	28/06/2022	829	S	S	S	ND	R	ND	S	S	S
1	A2162	09/01/2023	829	S	S	S	S	R	S	S	S	S
2	A693	28/04/2014	244	S	S	S	S	S	S	S	S	S
2	A709	02/12/2014	244	S	I	S	S	S	S	S	S	S
2	A700	23/04/2015	244	S	I	R	S	R	R	S	S	R
2	A725	11/12/2015	244	S	I	S	S	I	S	S	S	I
2	A743	16/12/2016	244	S	S	S	S	R	S	S	S	ND
2	A766	30/06/2017	244	S	S	S	S	R	S	S	S	S
3	A708	05/12/2014	2234	S	I	I	R	S	R	S	S	R
3	AP723	19/04/2016	2234	S	R	S	ND	I	ND	S	S	S
3	AM723	19/04/2016	2234	S	R	R	ND	I	ND	S	S	S
3	A747	13/09/2016	2234	S	S	S	S	S	S	S	S	S
3	A765	12/07/2017	2234	S	S	S	S	S	S	S	S	S
4	B972	11/07/2019	381	S	I	S	ND	S	ND	ND	S	ND
4	A961	08/03/2020	381	S	R	R	ND	R	ND	R	R	R
5	B699	23/01/2012	4052	R	R	R	R	I	R	S	S	R
6	A773	06/03/2019	2604	S	S	S	S	S	S	S	S	S
7	A776	18/05/2018	274	S	S	S	S	S	S	R	R	S
8	A928	06/07/2018	3426	S	S	S	S	S	S	S	S	S
9	A931	30/07/2019	Novel ST	S	S	S	ND	S	ND	S	S	S
10	A942	19/05/2018	254	S	S	R	ND	S	ND	S	S	S
11	A949	02/04/2018	155	S	S	S	ND	R	ND	S	S	S

^*a*^ AMK, amikacin; FEP, cefepime; CAZ, ceftazidime; CZA, ceftazidime/avibactam; CIP, ciprofloxacin; CT, ceftolozane/tazobactam; IMP, imipenem; MEM, meropenem; TZP, piperacillin/tazobactam; S, susceptible; I, intermediate; R, resistant; ND, not tested.

### Multi-locus sequence type, wgMLST, and single nucleotide polymorphism (SNP)-based phylogeny

Quality assessment metrics for the obtained assemblies are shown in Table S1.

**Fig 1:**
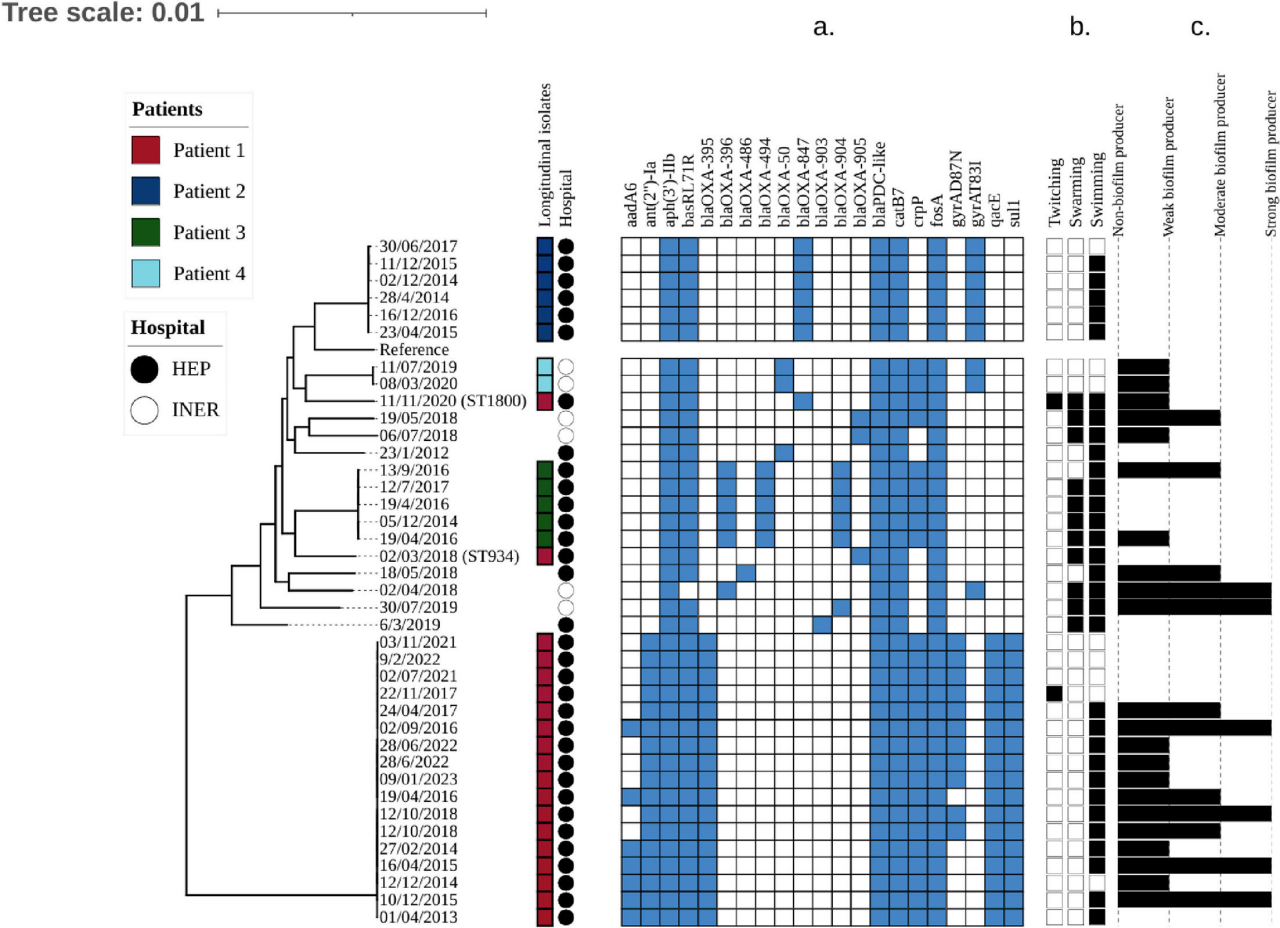
Midpoint-rooted SNP-based maximum-likelihood phylogenetic tree of included isolates; longitudinally collected isolates are represented with colors for patients 1–4, tree scale represents the number of substitutions per site. (**a**) Resistance genes presence/absence matrix; blue squares denote the presence of the genes specified on the top side of the matrix. (**b**) Results from the phenotypic evaluation of the three types of motilities exhibited by *P. aeruginosa*; black squares denote positivity for the type of motility specified on top. (**c**) Results from the quantitative assessment of biofilm production; length of bars represents the strength of biofilm production according to the criteria established by Stepanović et al. (42).

In patients with longitudinal isolates, a single sequence type (ST) prevailed over time; ST829 was identified in patient 1 isolates, ST244 was identified in patient 2 isolates, ST2234 for patient 3 isolates, and ST381 for patient 4 isolates. Notably, patient 1 isolates had a persistent (established) strain (ST829) and two sporadic strains (ST934 and ST1800; see Table 1), none of which shared MLST alleles with ST829. The SNP-based and wgMLST scheme phylogenetic trees confirmed that these strains were non-related, as they correspond to different clades in both phylogenies (Fig. S1). Similarities greater than 98% were observed in established strains, except in isolates from patient 2, in which the most recent isolate had a 92% distance from the earlier isolate (Fig. S1; Fig. 1). For each patient’s isolates, a similarity of approximately 20% was observed in the wgMLST tree, while they also formed independent clades in the SNP-based phylogeny. Additionally, the formation of clades is geographically independent, as isolates from both attention centers were distribute homogenously within the tree’s leaves (Fig. 1).

### Resistome analysis

In general, all strains harbored at least one allele of *bla*_OXA_ and *bla*_PDC_. The genes shared by all patients’ isolates—*aph*(*3’)-IIb*, *catB7*, and *fosA—*are associated with intrinsic resistance by antibiotic inactivation mechanisms. These genes are related to aminoglycoside, phenicol, and fosfomycin resistance, respectively.

Overall, genes harbored by all strains display a similar spectrum of action against cephalosporin and carbapenem antibiotics (Fig. 1; Table S2).

The patient 1 persistent isolates (ST829) harbored genes associated with resistance to antibiotics by efflux pumps (*qacE*; antiseptics), target site loss (*sul1*; sulfonamides), and target site alteration (*gyrA* D87N mutation; aminoglycosides). Two changes in the resistome of ST829 strains were observed over time: namely, the loss of the *aadA6* gene in 2017 and the acquisition of the previously mentioned *gyrA* mutation. The patient 1 non-related isolates (ST934 and ST1800) resistome was limited to *bla*_OXA_ and *bla*_PDC_ (Fig. 1). Isolates from patients 2 and 4 harbored the *gyrA* T83I mutation, which has been associated with increased resistance to aminoglycosides by target site alteration mechanism (Fig. 1).

### Virulome analysis

The virulome analysis using the EPISEQ CS software reveals an absence of genes related to flagellar structure and dynamics (*fleI/flag, fleP, flgK, flgL, filC, filD,* and *filS*), lipopolysaccharide (*hisFH* and *wbpABCDEGHIJL*), O-antigen biosynthesis (*wzx, wzy,* and *wzz*), type-IV pili biogenesis and structure (*pilA* and *pilB*), and pyoverdine metabolism (*fpvA, pvdEJP*) in most of the included isolates. Notably, this trend was not observed on isolates from patients 2 and 4 (ST234 and ST381, respectively; see Fig. S2 and S3). Patient 2 isolates harbored more virulence-associated genes than the other isolates, including *exoY* (exotoxin), *phzA1* (phenazine biosynthesis), *pvdA, pvdF* (pyoverdine metabolism), and *vgrG1b* (type-VI secretion system).

Conversely, biofilm-related genes (*muc*, *las*, *alg*, *pel,* and *psl* operons) were found to be conserved among all isolates, as were phenazines (*phz*)- and pyochelin (*pch*)-related genes. Permanent changes in the loss and gain of these genes were not observed in longitudinal isolates, as the absence and/or presence of genes followed an intermittent pattern over time. All the mentioned “absent” genes had a low bit-score ratio (<0.5) when analyzed by LS-BSR.

### Variant analysis

SNPs were the most frequently observed variant type, followed by insertions and deletions. When comparing the first vs the most recent isolate, the total number of mutations accumulated was 56 for patient 1 isolates over 10 years, 528 for patient 2 isolates over 3 years, and 39 for patient 3 isolates over 3 years. Considering the time of collection of isolates, mutation frequencies of 5, 176, and 13 mutations per year were obtained for patients 1, 2, and 3 isolates, respectively (Table S3). Relevant genes with changes over time for patient 1 isolates are *lasR*, *mucA*, and *algU*; and *xcpU*, *cdrA*, *mucA*, and *mutL* for patient 2 isolates. Isolates from patient 3 within these categories showed no mutational changes over time.

### Pangenome analysis

**Fig 2:**
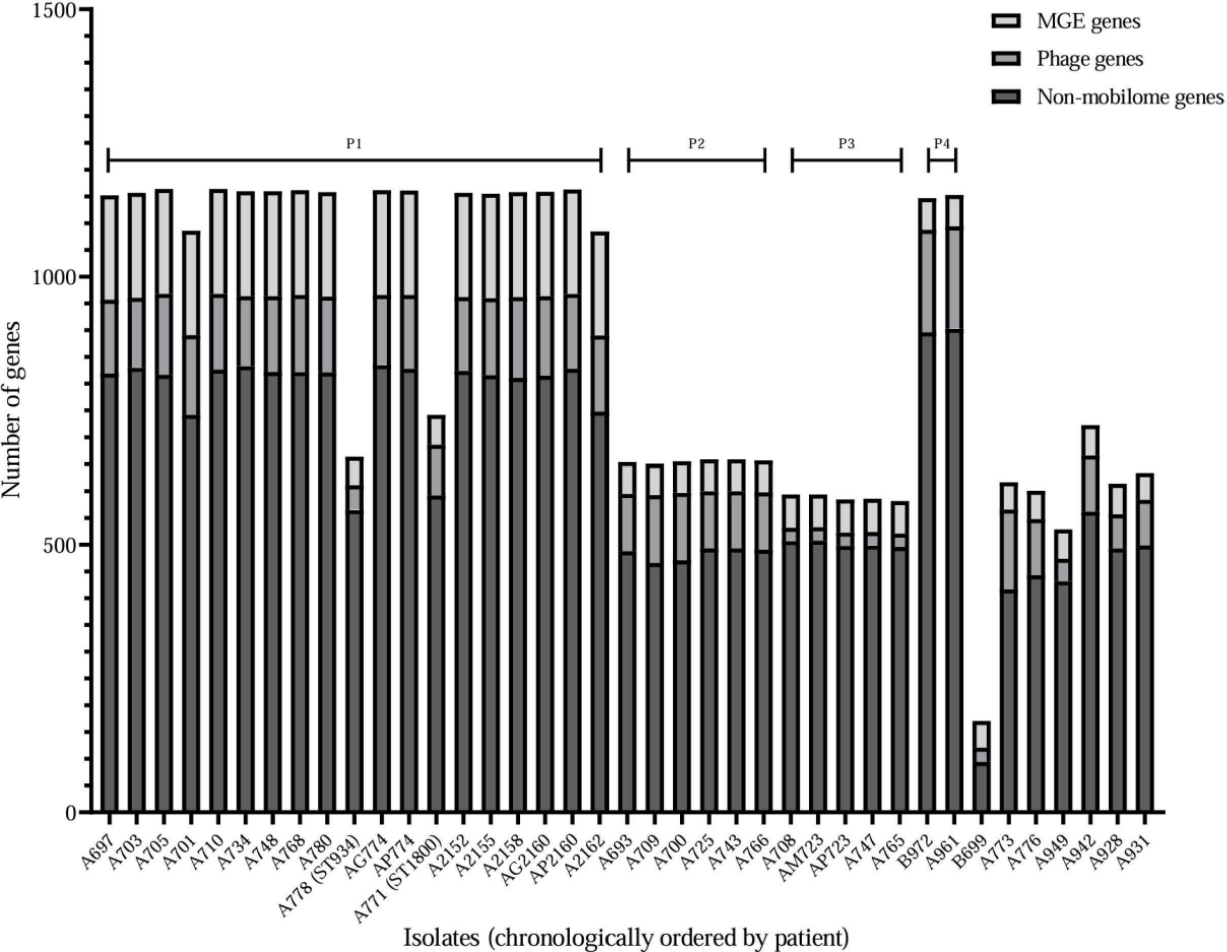
Accessory gene counts for each genome, and non-mobilome genes were calculated by subtracting the sum of the gene count contributions of the phage and MGEs from the calculated size of the accessory genome (P1, patient 1 isolates; P2, patient 2 isolates; P3, patient 3 isolates; P4, patient 4 isolates).

The core genome determined by mOTUpan (genes shared by 95% of the included isolates) was 5,258. Patient 1 established strain (ST829) had the largest accessory genome, ranging from 1,085 to 1,164 genes (Fig. 2a; Table S4); the average number of accessory genes for strains other than ST829 was 649 (171–742) genes. All isolates displayed a larger accessory genome than the PAO1 reference strain, except for ST4052 (patient 5 isolates), which consisted of 171 genes. Even though differences in the gene quantity were observed between isolates, the relative proportion of genes regarding COG20 functional annotation was similar (Fig. S4). The most frequent COG20 categories (average: >5% of the accessory genome in most isolates) were related to energy production, amino acid metabolism, lipid metabolism, transcription, translation, cell wall/membrane maintenance, inorganic ion transport, signal transduction, and mobilome (Fig. S4b).

It is noteworthy that an average of 10% of the pangenome content had either functional prediction only or an unknown function.

### Mobilome analysis

None of the putative plasmid sequences obtained from MOB-suite produced alignments against plasmid sequences, and thus its presence was discarded. For phage analysis, most of the identified sequences showed an integrity score from questionable to intact (Table S5). After conducting a similarity search of pyocins against putative phages using BLAST+ (Table S5), phage YMC11 displayed a positive result in strains harboring sequences for either F-type pyocins or both F- and R-type pyocins. Strains encoding only R-type pyocins displayed a positive result for phages phiCTX (NC_003278), Dobby (NC_048109), and Arya (NC_031048). Following pyocin filtering, PHASTEST identified phages B3 Pf1, F10, JBD-like (JBD24/25/67), phi297, PMG1 (hosted by *Pseudomonas* sp.), and S1 (hosted by *Stenotrophomonas* sp.) as the most similar hits. The number of phages by ST is shown in Table S6. Strains ST244, ST934, ST3426, and ST4052 harbored no phage sequences (apart from pyocins); strains ST155, ST254, ST1800, and the novel ST from patient 11 harbored one phage sequence; two phages were identified in ST274 and ST2604; and ST381 and ST829 were positive for three phage sequences.

Mobile genetic elements (MGEs) identified by ICEfinder are shown in Table S7; most of them being insertion sequences with no reported coding functions other than transposases. Patient 1 established strain (ST829) harbored 11 MGEs, seven of which are unique to this strain and include functions such as resistance to metals and biphenyl degradation; strain-specific MGEs are included in Table 2. Functions related to exotoxin A were identified in three insertion sequences (ISPa-2, ISPa-4, and ISPa-5), two of which were unique to ST244 (patient 2) and ST254 (patient 9).

**TABLE 2:** Strain-specific MGEs identified by MGEfinder; results were manually searched in the corresponding databases, and the specified function was obtained from each of these^*a*^

Strain	MGE	Type	Database	Accession ID	Associated functions
ST829 (patient 1; established)	ISPa11	IS	ISFinder	AE004697	Transposase
	ISPa41	IS	ISFinder	N/A	Transposase
	ISPa127	IS	ISFinder	N/A	Transposase
	ISPen2	IS	ISFinder	NC_008027	Transposase
	PAGI-5	ICE	ICEberg 3.0	ICEO_0000117	Mercury resistance
	Tn4656	Tn	The transposon registry	AB062597	Toluene degradation
	Tn4661	Tn	The transposon registry	AB375440	Transposase
ST244 (patient 2)	ISPa22	IS	ISFinder	NC_002516	Transposase
ST2234 (patient 3)	ISPa5	IS	ISFinder	U16785	Exotoxin A
	ISPa123	IS	ISFinder	CP041774	Transposase
	ISPa124	IS	ISFinder	CP041774.1	Transposase
ST381 (patient 4)	ISBcen18	IS	ISFinder	N/A	Transposase
	ISPa26	IS	ISFinder	EU000222	Transposase
	ISPa128	IS	ISFinder	CP054623.1	GNAT family N-acetyl transferase
	ISPpu17	IS	ISFinder	N/A	Transposase
ST155 (patient 8)	ISPa131	IS	ISFinder	CP041354	Transposase
ST254 (patient 9)	ISPa4	IS	ISFinder	U16785	Exotoxin A
ST3426 (patient 10)	ISPa97	IS	ISFinder	EU595745	Transposase

^*a*^ IS, insertion sequence; ICE, integrative conjugative element; Tn, transposon; N/A, not applicable

When considering the COG20 functional annotation, MGEs from all strains harbored functions mostly related to transcription (K), coenzyme metabolism (H), lipid metabolism (I), and signal transduction (T). Patient 1 ST829 MGEs harbored additional functions related to translation (J), extracellular structure (W), and replication/DNA repair (L). On the other hand, phages harbored fewer functions, which are mostly related to transcription (K), replication/DNA repair (L), and coenzyme metabolism (H). Both MGEs and phages harbor a large proportion of functional annotations related to the mobilome (X) and are mostly represented by the presence of transposases and structural proteins (Fig. S5 and S6).

### Antibiotic susceptibility testing

Strains showed an antibiotic-susceptible profile, with no multidrug-resistant isolates found with the highest resistance detected for ciprofloxacin (30%) (Table 1). Isolates from patient 1 displayed resistance to ciprofloxacin starting in 2016, and this resistance was maintained throughout the observation period. One early isolate from patient 1 (2014) displayed resistance to carbapenem, but this resistance was not observed in subsequent isolates. Additionally, three isolates showed resistance to amikacin (2021–2022). Both sporadic isolates from patient 1 (ST934 and ST1800) showed full susceptibility to the antibiotics evaluated. Similarly, early isolates from patients 2 and 4 (2014) did not display ciprofloxacin resistance, although later isolates did display such resistance. Conversely, isolates from patient 3 displayed an early antibiotic-resistant phenotype, but recent isolates did not display resistance. A tendency toward an increase in amikacin, cefepime, and ceftazidime minimum inhibitory concentration is observed for ST829 (patient 1 isolates) and ST244 (patient 2). Furthermore, patient 4 isolates (ST381) acquired resistance to cephalosporin and carbapenem antibiotics. Overall, most single-patient isolates displayed an antibiotic-susceptible profile, except for patient 5 strain (ST4052), which showed resistance to all the evaluated antibiotics except for the carbapenem class.

### Motility assays

Flagellum-dependent motility was frequent, whereas twitching motility was mostly absent in the isolates (Fig. 1). Swarming motility was observed in 28% of the isolates, all of which displayed swimming capacity. No increase or decrease was observed in longitudinally collected isolates in either type of motility, as they displayed stochastic behavior. A comparison of ST829 (patient 1 established isolate) with ST934 and ST1800 (patient 1 sporadic isolates) revealed that the former did not display swarming motility, whereas the sporadic isolates did.

### Elastolytic activity

**Fig 3:**
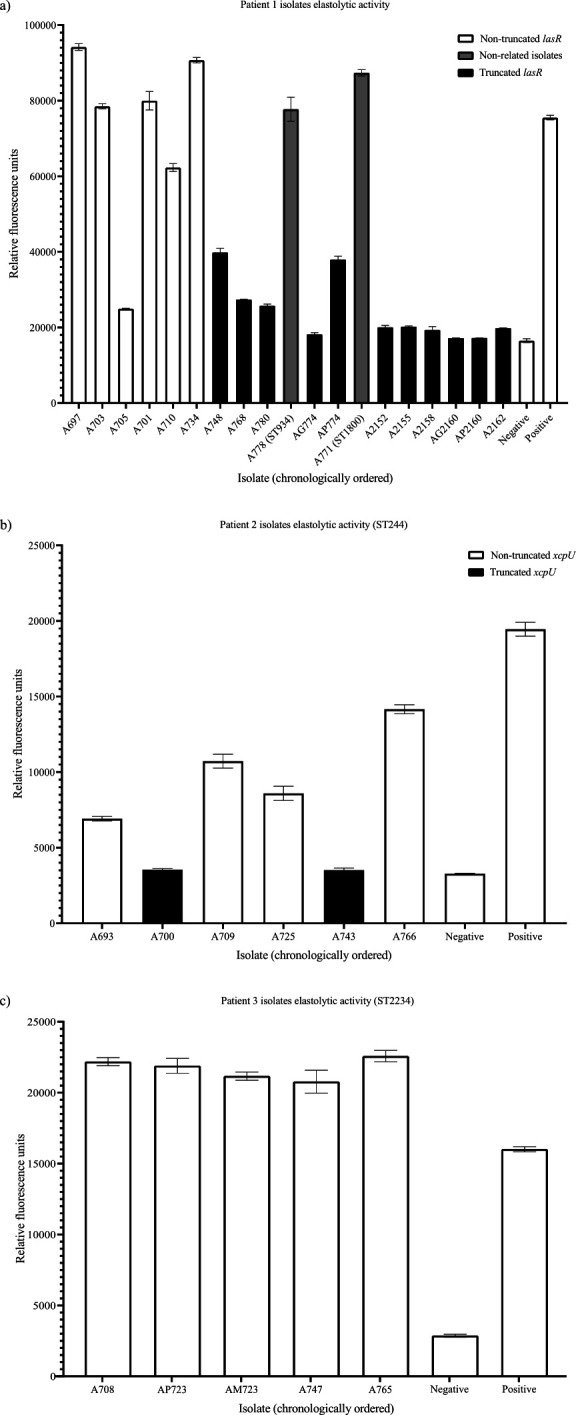
Semi-quantitative elastolytic activity assay results for longitudinal isolates. Black bars indicate isolates with truncated versions of the specified genes (*LasR* for patient 1 ST829, and *xcpU* for patient 2 ST244).

Secreted elastase was evaluated for longitudinal isolates (Fig. 3). A gradual decrease in activity was observed in ST829 (patient 1 established strain) until it was completely lost, while ST934 and ST1800 displayed high elastase activity, whereas, at the time point in which they were isolated, ST829 showed no activity.

Strain ST244 (patient 2) displayed a variable pattern over time, while ST2234 (patient 3) maintained the activity over time (Fig. 3). For ST829 (patient 1), a truncated *lasR* sequence was identified in 11/12 isolates that displayed a diminishment in elastolytic activity (isolate A748; 09/2016) onward. For ST244 (patient 2), truncated sequences of *xcpU* were identified in both isolates with decreased elastolytic activity. Finally, for ST2234 (patient 3), both previously mentioned genes displayed no significant mutations (see Fig. S7 for multiple sequence alignment of the mentioned genes).

### Growth rate

**Fig 4:**
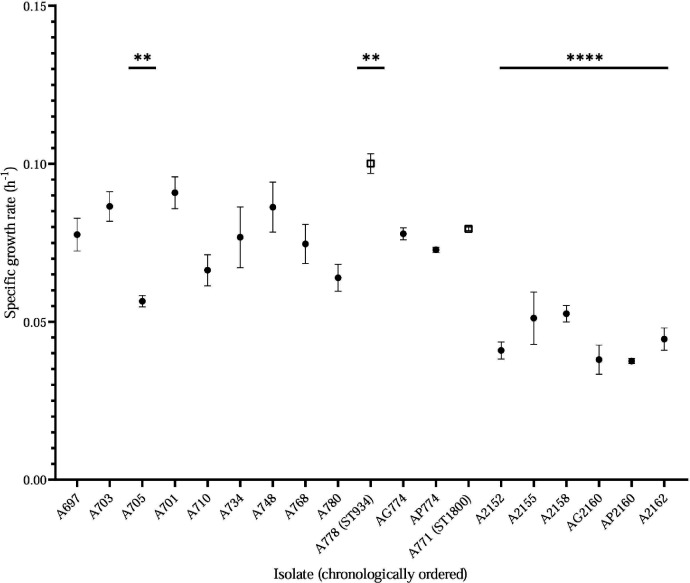
Patient 1 isolates’ specific growth rate, and non-related isolates are represented by squares; Dunnett’s test was used to compare all isolates vs the first one (**, P < 0.01; ****, *P* < 0.0001).

All isolates had a specific growth rate in the range of 0.04–0.1 h^−1^ (Table S8). For longitudinally collected isolates, ST829 (patient 1 established strain) displayed a reduction of the specific growth rate from approximately 0.09–0.04 h^−1^, which was statistically different on isolates after the year 2021 when compared to the first isolate (*P* < 0.001; Dunnett’s test), while ST934 (isolated on the year 2018) displayed a higher growth rate (Fig. 4). Conversely, isolates from patients 2 and 3 showed an intermittent pattern (Table S8).

### Biofilm production

Strains from patients 1 and 3 displayed a variable production capacity that followed no clear tendency, whereas all patient 2 isolates were non-producers (Fig. 1). This variable production was also seen in the individual isolates included in the sample.

## DISCUSSION

The MLST analysis suggests clonal persistence over time in patients with longitudinally collected isolates, and this trend is confirmed by wgMLST and SNP maximum-likelihood phylogenetic analysis. Every patient harbored a unique ST, most of which had few or no reports in the literature and PubMLST. Exceptions included ST244 (43–45) and ST381, both of which are associated with antimicrobial-resistant phenotypes (46–48), and ST274, which is considered a high-risk clone and was recently associated with CF-patient colonization (49).

A non-clonal distribution was observed between patients and attention centers, as each patient harbored a single clone, and these clones did not demonstrate a geographical relationship. Isolates from both centers were distributed evenly in different clades. This finding is aligned with previous reports that non-CF clinical isolates of *P. aeruginosa* follow a similar distribution pattern in Mexico (50).

Every strain harbored at least one allele of both *bla*_PDC_*_-_* and *bla*_OXA-type_ genes. *Pseudomonas*-derived cephalosporinases (*bla*_PDC_) are chromosomally encoded AmpC-type β-lactamases and have recently been described as one of the main β-lactam resistance mechanisms in the context of CF and tend to evolve toward a higher hydrolytic activity (51). However, it is noteworthy that even though ST244 (patient 2 isolates) and ST2234 (patient 3 isolates) share *bla*_PDC_ alleles, they display different susceptibility patterns for the β-lactams tested, suggesting that additional resistance mechanisms might be involved. Additionally, this finding contrasts with data generated in a previous Mexican study, in which clinical isolates from non-CF patients carried diverse carbapenemases, such as *bla*_VIM_*, bla*_IMP_*,* and *bla*_GES_ (50), which were not detected in the included isolates.

Based on the antibiotic susceptibility testing results, the acquisition of *gyrA* mutations may explain ciprofloxacin resistance in isolates that harbor these variants, as in the case of the ST829 simultaneous mutation and resistance gain in 2016. However, early isolates from patient 2 (ST244) harbored this mutation and displayed a susceptible profile. A similar case of no gene-phenotype correlation is seen in the *aadA6* gene; although it was lost in ST829, some subsequent isolates displayed resistance to amikacin. The mixed resistance to ciprofloxacin observed between isolates could be associated with the variable subtypes of *bla*_OXA_ and *bla*_PDC_ β-lactamases and the presence of variants.

These findings suggest a poor correlation between resistance genes and *in vitro* susceptibility testing; other factors, such as biofilm lifestyle, mucoid phenotypes, and decreased growth rate, may have a role in drug resistance (9, 16).

The virulome pattern of strains denotes a tendency to lack genes related to the flagellum, type-IV pili, O-antigen, lipopolysaccharide, and pyoverdine metabolism. Conversely, biofilm-related genes were conserved in all isolates included in this study. These findings correlate with the overall genomic profile observed in CF isolates, which is characterized by an evolution toward less virulent phenotypes (9, 10, 16). When comparing longitudinal isolates, patients 1 and 3 isolates display a lower frequency of virulence genes when compared to patient 2 strains. The patient 2 strain was detected to be ST244, which is widely distributed and associated with antimicrobial resistance phenotypes (44), which might play a role in the differences observed with the other strains.

Furthermore, genes related to phenazine biosynthesis (*phz*) displayed a high frequency among isolates that demonstrated polymorphic behavior when analyzed by LS-BSR (Fig. S3).

It has been reported that *P. aeruginosa* has a remarkable potential for competition with other bacterial species (52, 53). In this study, patient 1 established strain (ST829) harbored less virulence-associated genes when compared to the two sporadic strains from this patient (ST934 and ST1800); they also exhibited a greater elastolytic activity and faster growth rate *in vitro*. Even though these strains can be thought of as more virulent, they were not able to displace the stablished strain (ST829), which was capable of withstanding competition through an unexplored mechanism.

It is important to consider that even though EPISEQ CS and LS-BSR virulome analysis demonstrated an “absence” of the mentioned virulence genes, when each gene in MSA was analyzed, a great proportion of these were present with a great proportion of mismatches (*fleIP, flgKL, fleP, pvdEP, wbpAKL, wzz, HisF1H1*), opening the question as to whether these mutations have a functional effect on the coded proteins.

According to Camus et al., hypermutator strains tend to have a mutation rate of 2–350 SNPs per year, and all longitudinal isolates would be considered hypermutators according to these criteria (16). However, it is noteworthy that patient 2 isolates (ST244) demonstrated a higher mutation rate and a greater frequency of virulence-related genes than patient 1 isolates.

Pangenome analysis revealed a high proportion of genes related to energy production using different sources such as amino acids and lipids, as well as a high proportion of genes involved in cell wall/membrane homeostasis in the accessory genome. Few studies have included analysis of the accessory genome of strains in the context of CF. According to a study by Turner et al., the essential genes for establishing chronic infections in the CF-patient lung are mostly present within the core genome (54). This finding suggests that accessory genes are related to patient-specific microenvironments. However, mobile elements, such as prophages and pathogenicity islands, are frequent in this context and may play a role in the genetic diversification and establishment of infection (10, 16). These elements are thought to be enriched in the accessory genome (55), as found in this study.

Flagellum-dependent motility types were found to be conserved in most of the isolates, and twitching was practically absent. This finding is aligned with other studies that have found that twitching motility is not frequent in strains isolated from CF patients (21, 23–25). The absence of twitching could be attributed to the absence of *pilA* (56), which is also a common trait observed by Bianconi et al. (21). In the present study, strains exhibiting swimming motility also demonstrated swarming capabilities, but the reverse was not consistently true. No discernible trend indicating the loss or acquisition of these traits was observed among longitudinal isolates.

Two flagellum virulence gene patterns were observed among the study sample. In one pattern, the absence of genes was related to the outermost structures of the flagellum: the flagellin filament/cap (*fliCD*) and the apical portion of the hook (*flgK, flgL*). In the second pattern, the only missing gene was *fliR*, an integral component of the flagellum (57). However, this missing gene did not influence the swarming behavior of these strains, which suggests that additional mechanisms might be involved. A duplication event was observed in the most recent isolate from patient 2 (ST244, 2017) in *fliQ* (part of the basal body), which could be the cause of the loss of swimming capabilities of the strain. Additionally, the LS-BSR results indicate that missing genes identified by EPISEQ CS are present with a LS-BSR score of ≈0.5, which suggests that the gene is not completely lost but expressed as a truncated protein that still has function. This interpretation would explain the poor correlation between genotypic and phenotypic characteristics.

Generally, findings from comparable studies have indicated heterogeneity in the preservation of *in vitro* swimming or swarming motility. Notably, no definitive loss of these motility traits has been consistently observed; rather, they tend to be displayed intermittently, aligning with similar observations in previous studies (21, 23–25) and the current study. When comparing the accessory genome of patient 1 established isolates (ST829) to sporadic isolates (ST934 and ST1800), a greater metabolic versatility was found in the former.

Biofilm capacities of these strains displayed a variable behavior between isolates and strains, mostly between non-producers and weak-to-moderate producers, even though all biofilm-related genes were conserved in all isolates. This finding is notable because the biofilm lifestyle is strongly associated with chronic infections with *P. aeruginosa*, especially in CF patients (9, 10, 58, 59). Studies using methodologies like the one employed in the present work have reported similar results (25, 60, 61). Notably, no biofilm model can provide an exact representation of the *in vivo* characteristics of the CF lung microenvironment, such as the variable phenotypes expressed by the same strain (18, 62) or with other species (20, 63). This may explain the lack of correlation between phenotypic and molecular results for this phenotypic trait.

For the ST829 strain, non-synonymous mutations in the *algU/algT* regulatory gene (which stimulates the production of alginate) were detected in isolates with no biofilm production, except for isolate A697 (patient 1 first isolate; see Fig. S8), which is the only one that has a wild-type *mucA* gene and thus is non-mucoid. A recent study by Malhotra et al. (62) has demonstrated the important role of alginate in the development of biofilm in the absence of other matrix components such as psl and pel, and in the absence of the regulatory biofilm inductor c-di-GMP, which is in line with the current findings.

The tendency to exhibit slow growth rates was initially observed by Yang et al., in which *in situ* CF-derived isolates of *P. aeruginosa* exhibited a slower growth rate than reference strains PAO1 and PA14, a phenomenon associated with fitness advantages (14). Markussen et al. studied a single CF strain over 32 years and obtained similar results regardless of the origin of isolation (23). In our study, the persistent strain of patient 1 (ST 829) followed this tendency when comparing recent isolates to the ancestor isolate.

In elastase assays, an overtime reduction of elastolytic activity in established ST829 strain form patient 1 was observed, unlike the two sporadic strains (ST934 and ST1800). This decrease might be linked to a deletion in the *lasR* regulatory gene, as reported by Llanos et al. (15), who observed similar reductions in CF-patient isolates even when possessing a WT *lasB* sequence.

This study has some limitations, including the possible phenotypic changes associated with the preservation and maintenance of isolates (subcultures), especially when considering the existence of plasmids. Additionally, no patient management or clinical data were considered, all of which may impact the adaptation of strains. Finally, it is important to consider that multiple sublineages of the same strain of *P. aeruginosa* may coexist in the CF lung microenvironment; thus, the complex interaction between these sublineages and other strains or species may not be accurately represented.

### Conclusion

The genomic approaches and phenotypic characterization showed the persistence of *P. aeruginosa* isolates over time in patients with CF who had a longitudinal follow-up. A lower virulence profile was observed at the phenotypic level, reflected as a decrease in growth rate and elastolytic activity and the absence of twitching motility. On the genomic scale, established ST829 strain from patient 1 harbored a larger accessory genome and a much wider proportion of functions associated with mobilome elements, as well as fewer virulence factor-related genes. A tendency to lack genes related to pyoverdine, flagellum, pili, and O-antigen was observed, whereas those related to biofilm, phenazine, and pyochelin were conserved among isolates. Longitudinal isolates over 10 years demonstrated an adaptive phenotype.

## Data Availability

The raw sequencing reads are available in the National Center for Biotechnology Information (NCBI) short read archive under project number PRJNA1010856.

## SUPPLEMENTAL MATERIAL

Supplemental figures legends spectrum.01100-24-s0001.docx Legends for Supplemental Fig. S1 to S8.

Figure S1 spectrum.01100-24-s0002.tiff wgMLST phylogenetic tree.

Figure S2 spectrum.01100-24-s0003.tiff Absence/presence matrix of virulence factors genes.

Figure S3 spectrum.01100-24-s0004.tiff Large-scale BLAST bit-score ratio (LS-BSR) heatmap.

Figure S4 spectrum.01100-24-s0005.tiff (a) Total number of COG20 annotations of the accessory genome and (b) proportion of COG20 annotations from the accessory genome.

Figure S5 spectrum.01100-24-s0006.tiff (a) Total number of COG20 annotations of the identified phage sequences and (b) proportion of COG20 annotations from the identified phage sequences.

Figure S6 spectrum.01100-24-s0007.tiff (a) Total number of COG20 annotations of the identified mobile genetic elements and (b) proportion of COG20 annotations from the identified mobile genetic elements.

Figure S7 spectrum.01100-24-s0008.tiff Nucleotide multiple sequence alignment of (a) *LasR* and (b) *xcpU* sequences.

Figure S8 spectrum.01100-24-s0009.tiff Amino acid multiple sequence alignment of algU sequences.

Supplemental tables spectrum.01100-24-s0010.docx Tables S1 to S8.

ASM does not own the copyrights to Supplemental Material that may be linked to, or accessed through, an article. The authors have granted ASM a non-exclusive, world-wide license to publish the Supplemental Material files. Please contact the corresponding author directly for reuse.

## References

[1] Guo J, Garratt A, Hill A. 2022. Worldwide rates of diagnosis and effective treatment for cystic fibrosis. J Cyst Fibros 21:456–462. doi:10.1016/j.jcf.2022.01.009 35125294

[2] McBennett KA, Davis PB, Konstan MW. 2022. Increasing life expectancy in cystic fibrosis: advances and challenges. Pediatr Pulmonol 57 Suppl 1:S5–S12. doi:10.1002/ppul.25733 PMC9004282 34672432

[3] Plant BJ, Goss CH, Plant WD, Bell SC. 2013. Management of comorbidities in older patients with cystic fibrosis. Lancet Respir Med 1:164–174. doi:10.1016/S2213-2600(13)70025-0 24429097

[4] Dickinson KM, Collaco JM. 2021. Cystic fibrosis. Pediatr Rev 42:55–67. doi:10.1542/pir.2019-0212 33526571 PMC8972143

[5] De Boeck K. 2020. Cystic fibrosis in the year 2020: a disease with A new face. Acta Paediatr 109:893–899. doi:10.1111/apa.15155 31899933

[6] Simmonds NJ, Cullinan P, Hodson ME. 2009. Growing old with cystic fibrosis – The characteristics of long-term survivors of cystic fibrosis. Respir Med 103:629–635. doi:10.1016/j.rmed.2008.10.011 19022643

[7] Chen Q, Shen Y, Zheng J. 2021. A review of cystic fibrosis: basic and clinical aspects. Animal Model Exp Med 4:220–232. doi:10.1002/ame2.12180 34557648 PMC8446696

[8] Patient Registry Annual Data Report: Cystic Fibrosis Foundation. 2021. Available from: 2021 Annual Data Report (cff.org)

[9] Malhotra S, Hayes D, Wozniak DJ. 2019. Cystic fibrosis and Pseudomonas aeruginosa: the host-microbe interface. Clin Microbiol Rev 32:e00138-18. doi:10.1128/CMR.00138-18 31142499 PMC6589863

[10] Rossi E, La Rosa R, Bartell JA, Marvig RL, Haagensen JAJ, Sommer LM, Molin S, Johansen HK. 2021. Pseudomonas aeruginosa adaptation and evolution in patients with cystic fibrosis. Nat Rev Microbiol 19:331–342. doi:10.1038/s41579-020-00477-5 33214718

[11] Lund-Palau H, Turnbull AR, Bush A, Bardin E, Cameron L, Soren O, Wierre-Gore N, Alton E, Bundy JG, Connett G, Faust SN, Filloux A, Freemont P, Jones A, Khoo V, Morales S, Murphy R, Pabary R, Simbo A, Schelenz S, Takats Z, Webb J, Williams HD, Davies JC. 2016. Pseudomonas aeruginosa infection in cystic fibrosis: pathophysiological mechanisms and therapeutic approaches. Expert Rev Respir Med 10:685–697. doi:10.1080/17476348.2016.1177460 27175979

[12] Colque CA, Albarracín Orio AG, Feliziani S, Marvig RL, Tobares AR, Johansen HK, Molin S, Smania AM. 2020. Hypermutator Pseudomonas aeruginosa exploits multiple genetic pathways to develop multidrug resistance during long-term infections in the airways of cystic fibrosis patients. Antimicrob Agents Chemother 64:e02142-19. doi:10.1128/AAC.02142-19 32071060 PMC7179616

[13] Oliver A, Cantón R, Campo P, Baquero F, Blázquez J. 2000. High frequency of hypermutable Pseudomonas aeruginosa in cystic fibrosis lung infection. Science 288:1251–1254. doi:10.1126/science.288.5469.1251 10818002

[14] Yang L, Haagensen JAJ, Jelsbak L, Johansen HK, Sternberg C, Høiby N, Molin S. 2008. In situ growth rates and biofilm development of Pseudomonas aeruginosa populations in chronic lung infections. J Bacteriol 190:2767–2776. doi:10.1128/JB.01581-07 18156255 PMC2293235

[15] Llanos A, Achard P, Bousquet J, Lozano C, Zalacain M, Sable C, Revillet H, Murris M, Mittaine M, Lemonnier M, Everett M. 2023. Higher levels of Pseudomonas aeruginosa LasB elastase expression are associated with early-stage infection in cystic fibrosis patients. Sci Rep 13:14208. doi:10.1038/s41598-023-41333-9 37648735 PMC10468528

[16] Camus L, Vandenesch F, Moreau K. 2021. From genotype to phenotype: adaptations of Pseudomonas aeruginosa to the cystic fibrosis environment. Microb Genom 7:mgen000513. doi:10.1099/mgen.0.000513 33529147 PMC8190622

[17] La Rosa R, Rossi E, Feist AM, Johansen HK, Molin S. 2021. Compensatory evolution of Pseudomonas aeruginosa’s slow growth phenotype suggests mechanisms of adaptation in cystic fibrosis. Nat Commun 12:3186. doi:10.1038/s41467-021-23451-y 34045458 PMC8160344

[18] Malhotra S, Limoli DH, English AE, Parsek MR, Wozniak DJ. 2018. Mixed communities of mucoid and nonmucoid Pseudomonas aeruginosa exhibit enhanced resistance to host antimicrobials. MBio 9:e00275-18. doi:10.1128/mBio.00275-18 29588399 PMC5874919

[19] Lopes SP, Ceri H, Azevedo NF, Pereira MO. 2012. Antibiotic resistance of mixed biofilms in cystic fibrosis: impact of emerging microorganisms on treatment of infection. Int J Antimicrob Agents 40:260–263. doi:10.1016/j.ijantimicag.2012.04.020 22770521

[20] Price CE, Brown DG, Limoli DH, Phelan VV, O’Toole GA. 2020. Exogenous alginate protects Staphylococcus aureus from killing by Pseudomonas aeruginosa. J Bacteriol 202. doi:10.1128/JB.00559-19 PMC7099135 31792010

[21] Bianconi I, Jeukens J, Freschi L, Alcalá-Franco B, Facchini M, Boyle B, Molinaro A, Kukavica-Ibrulj I, Tümmler B, Levesque RC, Bragonzi A. 2015. Comparative genomics and biological characterization of sequential Pseudomonas aeruginosa isolates from persistent airways infection. BMC Genomics 16:1105. doi:10.1186/s12864-015-2276-8 26714629 PMC4696338

[22] Marvig RL, Dolce D, Sommer LM, Petersen B, Ciofu O, Campana S, Molin S, Taccetti G, Johansen HK. 2015. Within-host microevolution of Pseudomonas aeruginosa in Italian cystic fibrosis patients. BMC Microbiol 15:218. doi:10.1186/s12866-015-0563-9 26482905 PMC4612410

[23] Markussen T, Marvig RL, Gómez-Lozano M, Aanæs K, Burleigh AE, Høiby N, Johansen HK, Molin S, Jelsbak L. 2014. Environmental heterogeneity drives within-host diversification and evolution of Pseudomonas aeruginosa. MBio 5:e01592-14. doi:10.1128/mBio.01592-14 25227464 PMC4172072

[24] Cramer N, Klockgether J, Wrasman K, Schmidt M, Davenport CF, Tümmler B. 2011. Microevolution of the major common Pseudomonas aeruginosa clones C and PA14 in cystic fibrosis lungs. Environ Microbiol 13:1690–1704. doi:10.1111/j.1462-2920.2011.02483.x 21492363

[25] van Mansfeld R, de Been M, Paganelli F, Yang L, Bonten M, Willems R. 2016. Host evolution of the dutch high-prevalent Pseudomonas aeruginosa clone ST406 during chronic colonization of a patient with cystic fibrosis. PLOS ONE 11:e0158106. doi:10.1371/journal.pone.0158106 27337151 PMC4918941

[26] Wright MH, Adelskov J, Greene AC. 2017. Bacterial DNA extraction using individual enzymes and phenol/chloroform separation. J Microbiol Biol Educ 18:18.2.48. doi:10.1128/jmbe.v18i2.1348 PMC5577976 28861145

[27] Wick RR, Judd LM, Gorrie CL, Holt KE. 2017. Unicycler: resolving bacterial genome assemblies from short and long sequencing reads. PLOS Comput Biol 13:e1005595. doi:10.1371/journal.pcbi.1005595 28594827 PMC5481147

[28] Parks DH, Imelfort M, Skennerton CT, Hugenholtz P, Tyson GW. 2015. CheckM: assessing the quality of microbial genomes recovered from isolates, single cells, and metagenomes. Genome Res 25:1043–1055. doi:10.1101/gr.186072.114 25977477 PMC4484387

[29] Gurevich A, Saveliev V, Vyahhi N, Tesler G. 2013. QUAST: quality assessment tool for genome assemblies. Bioinformatics 29:1072–1075. doi:10.1093/bioinformatics/btt086 23422339 PMC3624806

[30] Seemann T. 2015. Snippy: fast bacterial variant calling from NGS reads

[31] Stamatakis A. 2014. RAxML version 8: a tool for phylogenetic analysis and post-analysis of large phylogenies. Bioinformatics 30:1312–1313. doi:10.1093/bioinformatics/btu033 24451623 PMC3998144

[32] Sahl JW, Caporaso JG, Rasko DA, Keim P. 2014. The large-scale blast score ratio (LS-BSR) pipeline: a method to rapidly compare genetic content between bacterial genomes. PeerJ 2:e332. doi:10.7717/peerj.332 24749011 PMC3976120

[33] Katoh K, Toh H. 2008. Recent developments in the MAFFT multiple sequence alignment program. Brief Bioinformatics 9:286–298. doi:10.1093/bib/bbn013 18372315

[34] Seemann T. 2014. Prokka: rapid prokaryotic genome annotation. Bioinformatics 30:2068–2069. doi:10.1093/bioinformatics/btu153 24642063

[35] Eren AM, Kiefl E, Shaiber A, Veseli I, Miller SE, Schechter MS, Fink I, Pan JN, Yousef M, Fogarty EC, et al.. 2021. Community-led, integrated, reproducible multi-omics with anvi’o. Nat Microbiol 6:3–6. doi:10.1038/s41564-020-00834-3 33349678 PMC8116326

[36] Buck M, Mehrshad M, Bertilsson S. 2022. mOTUpan: a robust Bayesian approach to leverage metagenome-assembled genomes for core-genome estimation. NAR Genom Bioinform 4:lqac060. doi:10.1093/nargab/lqac060 35979445 PMC9376867

[37] Wishart DS, Han S, Saha S, Oler E, Peters H, Grant JR, Stothard P, Gautam V. 2023. PHASTEST: faster than PHASTER, better than PHAST. Nucleic Acids Res 51:W443–W450. doi:10.1093/nar/gkad382 37194694 PMC10320120

[38] Johansson MHK, Bortolaia V, Tansirichaiya S, Aarestrup FM, Roberts AP, Petersen TN. 2021. Detection of mobile genetic elements associated with antibiotic resistance in Salmonella enterica using a newly developed web tool: MobileElementFinder. J Antimicrob Chemother 76:101–109. doi:10.1093/jac/dkaa390 33009809 PMC7729385

[39] Robertson J, Nash JHE. 2018. MOB-suite: software tools for clustering, reconstruction and typing of plasmids from draft assemblies. Microb Genom 4:e000206. doi:10.1099/mgen.0.000206 30052170 PMC6159552

[40] Camacho C, Coulouris G, Avagyan V, Ma N, Papadopoulos J, Bealer K, et al.. 2009. BLAST+: architecture and applications. BMC Bioinformatics 10:421. 20003500 10.1186/1471-2105-10-421 PMC2803857

[41] Filloux A. 2014. Pseudomonas: methods and protocols, p 816. Humana Press, New York.

[42] Stepanović S, Vuković D, Dakić I, Savić B, Švabić-Vlahović M. 2000. A modified microtiter-plate test for quantification of staphylococcal biofilm formation. J Microbiol Methods 40:175–179. 10699673 10.1016/s0167-7012(00)00122-6

[43] Pérez-vázquez M, Sola-campoy PJ, Zurita ÁM, Ávila A, Gómez-bertomeu F, Solís S, López-urrutia L, Gónzalez-barberá EM, Cercenado E, Bautista V, Lara N, Aracil B, Oliver A, Campos J, Oteo-iglesias J. 2020. Carbapenemase-producing Pseudomonas aeruginosa in Spain: interregional dissemination of the high-risk clones ST175 and ST244 carrying bla_VIM-2_, bla_VIM-1_, bla_IMP-8_, bla_VIM-20_ and bla_KPC-2_. Int J Antimicrob Agents 56:106026. doi:10.1016/j.ijantimicag.2020.106026 32450200

[44] Chen Y, Sun M, Wang M, Lu Y, Yan Z. 2014. Dissemination of IMP-6-producing Pseudomonas aeruginosa ST244 in multiple cities in China. Eur J Clin Microbiol Infect Dis 33:1181–1187. doi:10.1007/s10096-014-2063-5 24500601

[45] Fang Y, Baloch Z, Zhang W, Hu Y, Zheng R, Song Y, Tai W, Xia X. 2022. Emergence of carbapenem-resistant ST244, ST292, and ST2446 Pseudomonas aeruginosa clones in burn patients in Yunnan Province. Infect Drug Resist 15:1103–1114. doi:10.2147/IDR.S353130 35321081 PMC8935738

[46] Patil S, Chen X, Mai H, Lian M, Lopes BS, Liu S, Wen F. 2022. Genomic epidemiology and mechanisms of antibiotic resistance in Pseudomonas aeruginosa isolated from children’s hospital in Shenzhen, China. In Review. doi:10.21203/rs.3.rs-2090554/v1

[47] Merradi M, Kassah-Laouar A, Ayachi A, Heleili N, Menasria T, Hocquet D, Cholley P, Sauget M. 2019. Occurrence of VIM-4 metallo-β-lactamase-producing Pseudomonas aeruginosa in an Algerian hospital. J Infect Dev Ctries 13:284–290. doi:10.3855/jidc.10679 32045372

[48] Patil S, Chen X, Dong S, Mai H, Lopes BS, Liu S, Wen F. 2023. Resistance genomics and molecular epidemiology of high-risk clones of ESBL-producing Pseudomonas aeruginosa in young children. Front Cell Infect Microbiol 13:1168096. doi:10.3389/fcimb.2023.1168096 37293207 PMC10244630

[49] Chichón G, López M, de Toro M, Ruiz-Roldán L, Rojo-Bezares B, Sáenz Y. 2023. Spread of Pseudomonas aeruginosa ST274 clone in different niches: resistome, virulome, and phylogenetic relationship. Antibiotics (Basel) 12:1561. doi:10.3390/antibiotics12111561 37998763 PMC10668709

[50] Garza-Ramos U, Rodríguez-Medina N, Córdova-Fletes C, Rubio-Mendoza D, Alonso-Hernández CJ, López-Jácome LE, Morfín-Otero R, Rodríguez-Noriega E, Rojas-Larios F, Vázquez-Larios MDR, et al.. 2023. Whole genome analysis of Gram-negative bacteria using the EPISEQ CS application and other bioinformatic platforms. J Glob Antimicrob Resist 33:61–71. doi:10.1016/j.jgar.2023.02.026 36878463

[51] Colque CA, Albarracín Orio AG, Tomatis PE, Dotta G, Moreno DM, Hedemann LG, Hickman RA, Sommer LM, Feliziani S, Moyano AJ, Bonomo RA, K Johansen H, Molin S, Vila AJ, Smania AM. 2022. Longitudinal evolution of the Pseudomonas-derived cephalosporinase (PDC) structure and activity in a cystic fibrosis patient treated with β-lactams. MBio 13:e0166322. doi:10.1128/mbio.01663-22 36073814 PMC9600753

[52] Oluyombo O, Penfold CN, Diggle SP. 2019. Competition in biofilms between cystic fibrosis isolates of Pseudomonas aeruginosa is shaped by R-pyocins. MBio 10:e01828-18. doi:10.1128/mBio.01828-18 30696740 PMC6355985

[53] Scholl D. 2017. Phage tail-like bacteriocins. Annu Rev Virol 4:453–467. doi:10.1146/annurev-virology-101416-041632 28961412

[54] Turner KH, Wessel AK, Palmer GC, Murray JL, Whiteley M. 2015. Essential genome of Pseudomonas aeruginosa in cystic fibrosis sputum. Proc Natl Acad Sci USA 112:4110–4115. doi:10.1073/pnas.1419677112 25775563 PMC4386324

[55] Gabrielaite M, Johansen HK, Molin S, Nielsen FC, Marvig RL. 2020. Gene loss and acquisition in lineages of Pseudomonas aeruginosa evolving in cystic fibrosis patient airways. MBio 11:e02359-20. doi:10.1128/mBio.02359-20 33109761 PMC7593970

[56] Craig L, Forest KT, Maier B. 2019. Type IV pili: dynamics, biophysics and functional consequences. Nat Rev Microbiol 17:429–440. doi:10.1038/s41579-019-0195-4 30988511

[57] 2022. Pseudomonas aeruginosa: biology, pathogenesis and control strategies. Springer International Publishing, Cham.

[58] Høiby N, Ciofu O, Bjarnsholt T. 2010. Pseudomonas aeruginosa biofilms in cystic fibrosis. Future Microbiol 5:1663–1674. doi:10.2217/fmb.10.125 21133688

[59] Morris AJ, Jackson L, Cw Yau Y, Reichhardt C, Beaudoin T, Uwumarenogie S, Guttman KM, Lynne Howell P, Parsek MR, Hoffman LR, Nguyen D, DiGiandomenico A, Guttman DS, Wozniak DJ, Waters VJ. 2021. The role of Psl in the failure to eradicate Pseudomonas aeruginosa biofilms in children with cystic fibrosis. NPJ Biofilms Microbiomes 7:63. doi:10.1038/s41522-021-00234-3 34349133 PMC8338932

[60] Perez LRR, Costa MCN, Freitas ALP, Barth AL. 2011. Evaluation of biofilm production by Pseudomonas aeruginosa isolates recovered from cystic fibrosis and non-cystic fibrosis patients. Braz J Microbiol 42:476–479. doi:10.1590/S1517-838220110002000011 24031658 PMC3769813

[61] Smith EE, Buckley DG, Wu Z, Saenphimmachak C, Hoffman LR, D’Argenio DA, Miller SI, Ramsey BW, Speert DP, Moskowitz SM, Burns JL, Kaul R, Olson MV. 2006. Genetic adaptation by Pseudomonas aeruginosa to the airways of cystic fibrosis patients. Proc Natl Acad Sci U S A 103:8487–8492. doi:10.1073/pnas.0602138103 16687478 PMC1482519

[62] Jacobs HM, O’Neal L, Lopatto E, Wozniak DJ, Bjarnsholt T, Parsek MR. 2022. Mucoid Pseudomonas aeruginosa can produce calcium-gelled biofilms independent of the matrix components Psl and CdrA. J Bacteriol 204:e0056821. doi:10.1128/jb.00568-21 35416688 PMC9112934

[63] Vandeplassche E, Sass A, Lemarcq A, Dandekar AA, Coenye T, Crabbé A. 2019. In vitro evolution of Pseudomonas aeruginosa AA2 biofilms in the presence of cystic fibrosis lung microbiome members. Sci Rep 9:12859. 31492943 10.1038/s41598-019-49371-y PMC6731285

