# Supplementary material for: Genomic and phenotypic characterization of Pseudomonas aeruginosa isolates from two Mexican cystic fibrosis attention centers
Source: Microbiol Spectr. 2024 Oct 23;12(12):e01100-24. doi: 10.1128/spectrum.01100-24 (PMC11619361; doi:10.1128/spectrum.01100-24)
Supplement: Supplemental figures legends — Legends for Supplemental Fig. S1 to S8. [file spectrum.01100-24-s0001.docx]

**Figure S1.** wgMLST phylogenetic tree obtained from the EpiSeq platform. The scale represents the similarity between strains.

**Figure S2.** Absence/presence matrix of virulence factors genes obtained from the EpiSeq platform and midpoint-rooted maximum-likelihod phylogenetic tree of the included isolates. Blue squares indicate the presence of the corresponding gene on top, tree scale represents substitutions per site (HEP: Hospital de Especialidades Pediátricas; INER: Instituto Nacional de Enfermedades Respiratorias).

**Figure S3.** Large-scale BLAST bit-score ratio (LS-BSR) heatmap of virulence factors genes and midpoint-rooted maximum-likelihod phylogenetic tree of the included isolates. Virulence factors of the PAO1 strain were used as referemce. A value of 1 (blue)indicates 100% similarity and coverage, value of 0 (red) indicates an absence of the gene indicated on top. Tree scale represents substitutions per site (HEP: Hospital de Especialidades Pediátricas; INER: Instituto Nacional de Enfermedades Respiratorias).

**Figure S4.** (a) total number of COG20 annotations of the accessory genome for each of the included isolates and (b) proportion of COG20 annotations from the accessory genome, calculated by considering the total number of functional annotations as 100% for each (P1: patient 1 isolates; P2: patient 2 isolates; P3: patient 3 isolates; P4: patient 4 isolates).

**Figure S5.** (a) total number of COG20 annotations of the identified phage sequences for each of the included isolates and (b) proportion of COG20 annotations from the identified phage sequences, calculated by considering the total number of functional annotations as 100% for each (P1: patient 1 isolates; P2: patient 2 isolates; P3: patient 3 isolates; P4: patient 4 isolates).

**Figure S6.** (a) total number of COG20 annotations of the identified mobile genetic elements for each of the included isolates and (b) proportion of COG20 annotations from the identified mobile genetic elements, calculated by considering the total number of functional annotations as 100% for each (P1: patient 1 isolates; P2: patient 2 isolates; P3: patient 3 isolates; P4: patient 4 isolates).

**Figure S7.** (a) Nucleotide multiple sequence alignment of *lasR* sequences from patient 1 isolates using the PAO1 gene as a reference (top), isolates are chronologically ordered from top (oldest) to bottom and (b) nucleotide multiple sequence alignment of *xcpU* sequences from patient 2 isolates using the PAO1 gene as a reference (top), isolates are chronologically ordered from top (oldest) to bottom. The genomic coordinates of each sequence are specified.

**Figure S8.** Aminoacid multiple sequence alignment of *algU* sequences from patient 1 isolates using the PAO1 gene as a reference (top), isolates are chronologically ordered from top (oldest) to bottom.
