# Supplementary figures and images for: Genomic and phenotypic characterization of Pseudomonas aeruginosa isolates from two Mexican cystic fibrosis attention centers
Source: Microbiol Spectr. 2024 Oct 23;12(12):e01100-24. doi: 10.1128/spectrum.01100-24 (PMC11619361; doi:10.1128/spectrum.01100-24)

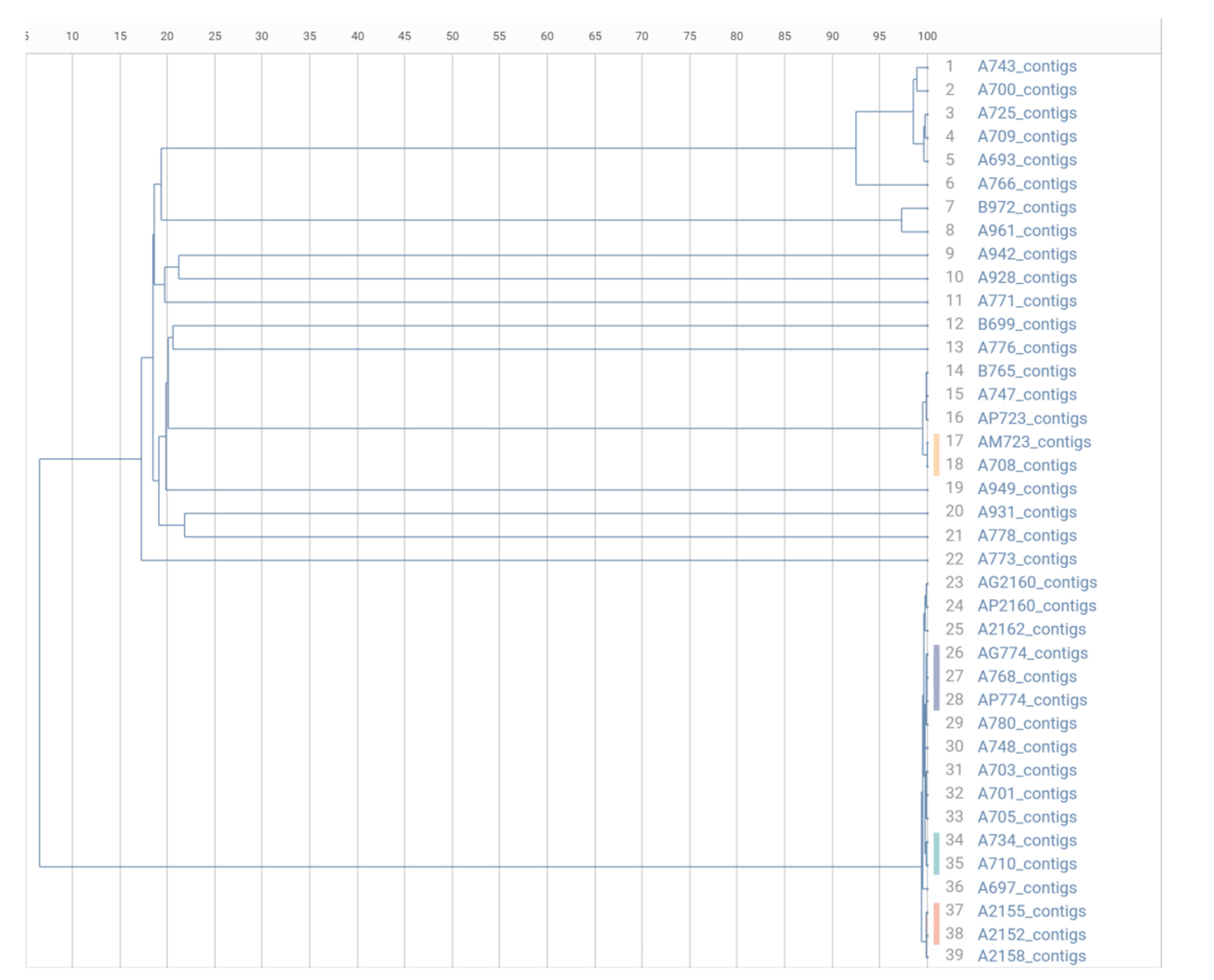

Supplement: Figure S1 — wgMLST phylogenetic tree. [file spectrum.01100-24-s0002.tiff]

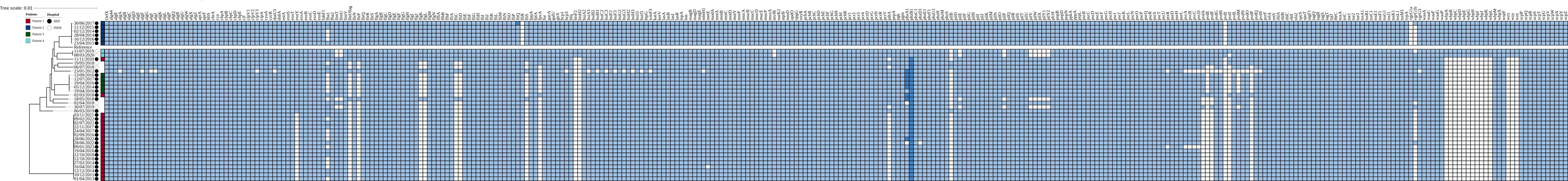

Supplement: Figure S2 — Absence/presence matrix of virulence factors genes. [file spectrum.01100-24-s0003.tiff]

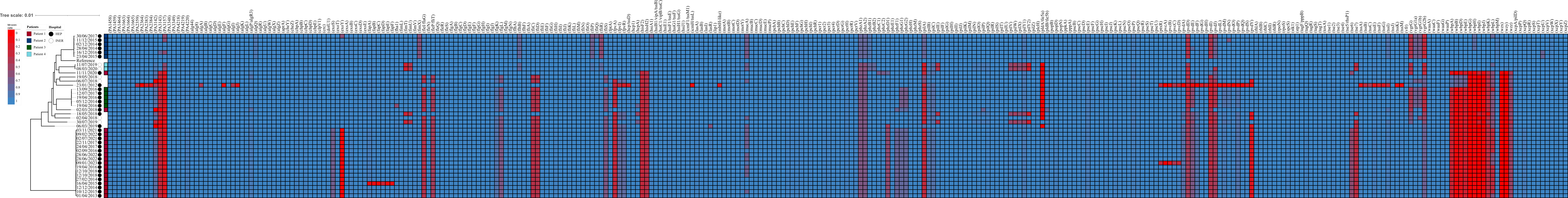

Supplement: Figure S3 — Large-scale BLAST bit-score ratio (LS-BSR) heatmap. [file spectrum.01100-24-s0004.tiff]
