# Supplementary material for: Genomic and phenotypic characterization of Pseudomonas aeruginosa isolates from two Mexican cystic fibrosis attention centers
Source: Microbiol Spectr. 2024 Oct 23;12(12):e01100-24. doi: 10.1128/spectrum.01100-24 (PMC11619361; doi:10.1128/spectrum.01100-24)
Supplement: Supplemental tables — Tables S1 to S8. [file spectrum.01100-24-s0010.docx]

| **Table S1. Genome-assembly quality metrics obtained by the QUAST software.** | | | | | | | | | | | |  |
| --- | --- | --- | --- | --- | --- | --- | --- | --- | --- | --- | --- | --- |
|  |  |  |  |  |  |  |  |  |  |  |  |  |
| Isolate | Isolation date | Patient | contigs | Largest contig | Total length | GC (%) | N50 | N90 | L50 | L90 | # N's per 100 kbp |  |
| A697 | 01/04/2013 | 1 | 80 | 1132587 | 6850966 | 66.08 | 370227 | 70647 | 6 | 21 | 0 |  |
| A703 | 27/02/2014 | 1 | 81 | 1132305 | 6850509 | 66.08 | 370227 | 70647 | 6 | 22 | 0 |  |
| A705 | 12/12/2014 | 1 | 81 | 1132305 | 6850452 | 66.08 | 370227 | 65713 | 6 | 23 | 0 |  |
| A701 | 16/04/2015 | 1 | 77 | 1132305 | 6772691 | 66.07 | 370227 | 81631 | 6 | 21 | 0 |  |
| A710 | 10/12/2015 | 1 | 82 | 1132305 | 6850501 | 66.08 | 312222 | 65713 | 6 | 24 | 0 |  |
| A734 | 19/04/2016 | 1 | 80 | 1132305 | 6850486 | 66.08 | 370227 | 65713 | 6 | 22 | 0 |  |
| A748 | 02/09/2016 | 1 | 82 | 1132702 | 6850906 | 66.08 | 370227 | 65713 | 6 | 24 | 0 |  |
| A768 | 24/04/2017 | 1 | 80 | 1132769 | 6850089 | 66.08 | 370227 | 70647 | 6 | 22 | 0 |  |
| A780 | 22/11/2017 | 1 | 80 | 1132763 | 6850067 | 66.08 | 370227 | 65713 | 6 | 22 | 0 |  |
| A778 | 02/03/2018 | 1 | 88 | 820877 | 6355578 | 66.45 | 395152 | 76231 | 6 | 18 | 0 |  |
| AG774 | 12/10/2018 | 1 | 83 | 1132735 | 6849116 | 66.08 | 370284 | 65716 | 6 | 22 | 0 |  |
| AP774 | 12/10/2018 | 1 | 84 | 1132735 | 6848547 | 66.08 | 370227 | 62375 | 6 | 23 | 0 |  |
| A771 | 11/11/2020 | 1 | 76 | 804143 | 6409989 | 66.31 | 300057 | 71050 | 7 | 23 | 0 |  |
| A2152 | 02/07/2021 | 1 | 84 | 916662 | 6850430 | 66.08 | 305475 | 69405 | 7 | 24 | 0 |  |
| A2155 | 03/11/2021 | 1 | 83 | 916662 | 6850343 | 66.08 | 305475 | 70647 | 7 | 23 | 0 |  |
| A2158 | 09/02/2022 | 1 | 85 | 916674 | 6850553 | 66.08 | 305478 | 65716 | 7 | 25 | 0 |  |
| AG2160 | 28/06/2022 | 1 | 80 | 1132762 | 6850042 | 66.08 | 370227 | 65713 | 6 | 22 | 0 |  |
| AP2160 | 28/06/2022 | 1 | 81 | 1132762 | 6848622 | 66.08 | 370227 | 65713 | 6 | 22 | 0 |  |
| A2162 | 09/01/2023 | 1 | 81 | 1132743 | 6761847 | 66.04 | 370345 | 65713 | 6 | 21 | 0 |  |
| A693 | 28/04/2014 | 2 | 63 | 1061198 | 6354201 | 66.22 | 384420 | 76484 | 5 | 18 | 0 |  |
| A709 | 02/12/2014 | 2 | 62 | 1061190 | 6353434 | 66.22 | 481462 | 76484 | 5 | 17 | 0 |  |
| A700 | 23/04/2015 | 2 | 63 | 1061171 | 6353035 | 66.22 | 481446 | 76484 | 5 | 17 | 0 |  |
| A725 | 11/12/2015 | 2 | 65 | 826095 | 6353986 | 66.22 | 467545 | 68439 | 6 | 19 | 0 |  |
| A743 | 16/12/2016 | 2 | 61 | 1061168 | 6353255 | 66.22 | 481451 | 76483 | 5 | 17 | 0 |  |
| A766 | 30/06/2017 | 2 | 55 | 1061203 | 6354746 | 66.22 | 481675 | 89953 | 5 | 16 | 0 |  |
| A708 | 05/12/2014 | 3 | 61 | 799808 | 6325204 | 66.49 | 473020 | 86744 | 5 | 18 | 0 |  |
| AM723 | 19/04/2016 | 3 | 64 | 799808 | 6324298 | 66.49 | 370830 | 72786 | 6 | 20 | 0 |  |
| AP723 | 19/04/2016 | 3 | 59 | 799812 | 6318670 | 66.49 | 473019 | 86744 | 5 | 18 | 0 |  |
| A747 | 13/09/2016 | 3 | 60 | 799812 | 6318504 | 66.49 | 370830 | 86891 | 6 | 18 | 0 |  |
| A765 | 12/07/2017 | 3 | 57 | 799811 | 6317873 | 66.49 | 473019 | 76091 | 5 | 18 | 0 |  |
| B972 | 11/07/2019 | 4 | 92 | 746159 | 6789050 | 66.08 | 256730 | 68339 | 9 | 26 | 0 |  |
| A961 | 08/03/2020 | 4 | 93 | 746158 | 6779633 | 66.09 | 253998 | 68340 | 9 | 26 | 0 |  |
| B699 | 23/01/2012 | 5 | 37 | 1011849 | 5830331 | 66.55 | 643010 | 106216 | 4 | 11 | 0 |  |
| A773 | 06/03/2019 | 6 | 49 | 853506 | 6355780 | 66.45 | 457304 | 196895 | 5 | 14 | 0 |  |
| A776 | 18/05/2018 | 7 | 58 | 661957 | 6295156 | 66.53 | 409892 | 90825 | 6 | 20 | 0 |  |
| A949 | 02/04/2018 | 8 | 65 | 910815 | 6300065 | 66.49 | 371313 | 76113 | 6 | 19 | 0 |  |
| A942 | 19/05/2018 | 9 | 66 | 659625 | 6421536 | 66.41 | 371758 | 84348 | 7 | 21 | 0 |  |
| A928 | 06/07/2018 | 10 | 45 | 902247 | 6337723 | 66.44 | 765925 | 108348 | 4 | 14 | 0 |  |
| A931 | 30/07/2019 | 11 | 61 | 666645 | 6344619 | 66.42 | 351781 | 90151 | 7 | 20 | 0 |  |

| **Table S2. Resistance genes identified by the EpiSeq software and its corresponding prevalence and spectrum of action, obtained from the Comprehensive Antibiotic Resistance Database (CARD).** | | |  |
| --- | --- | --- | --- |
|  |  |  |  |
| Gene | Prevalence on NCBI assemblies | Predicted spectrum of action |  |
| OXA-395 | 8% | cephalosporin, carbapenems |  |
| OXA-396 | 6% | cephalosporin, carbapenems |  |
| OXA-486 | 11% | cephalosporin, carbapenems |  |
| OXA-494 | 13% | cephalosporin, carbapenems |  |
| OXA-50 | 20% | cephalosporin, carbapenems |  |
| OXA-847 | 6% | cephalosporin, carbapenems |  |
| OXA-847 | 6% | cephalosporin, carbapenems |  |
| OXA-903 | 2% | cephalosporin, carbapenems |  |
| OXA-904 | 3% | cephalosporin, carbapenems |  |
| OXA-905 | 1% | cephalosporin, carbapenems |  |
| PDC-114 | 0% | cephalosporin, monobactam and carbapenems |  |
| PDC-14 | 0% | cephalosporin, monobactam and carbapenems |  |
| PDC-146 | 0% | cephalosporin, monobactam and carbapenems |  |
| PDC-162 | 0% | cephalosporin, monobactam and carbapenems |  |
| PDC-164 | 0% | cephalosporin, monobactam and carbapenems |  |
| PDC-176 | unknown | cephalosporin and carbapenems |  |
| PDC-183 | 0% | cephalosporin, monobactam and carbapenems |  |
| PDC-191 | unknown | cephalosporin and carbapenems |  |
| PDC-192 | unknown | cephalosporin, monobactam and carbapenems |  |
| PDC-212 | 0% | cephalosporin, monobactam and carbapenems |  |
| PDC-227 | 0% | cephalosporin, monobactam and carbapenems |  |
| PDC-272 | unknown | cephalosporin, monobactam and carbapenems |  |
| PDC-279 | 0% | cephalosporin, monobactam and carbapenems |  |
| PDC-3 | 20% | cephalosporin, monobactam and carbapenems |  |
| PDC-303 | 0% | cephalosporin, monobactam and carbapenems |  |
| PDC-31 | 2% | cephalosporin, monobactam and carbapenems |  |
| PDC-314 | 0% | cephalosporin, monobactam and carbapenems |  |
| PDC-335 | 0% | cephalosporin, monobactam and carbapenems |  |
| PDC-363 | 0% | cephalosporin, monobactam and carbapenems |  |
| PDC-366 | 0% | cephalosporin, monobactam and carbapenems |  |
| PDC-375 | 0% | cephalosporin, monobactam and carbapenems |  |
| PDC-380 | 0% | cephalosporin, monobactam and carbapenems |  |
| PDC-415 | 0% | cephalosporin, monobactam and carbapenems |  |
| PDC-423 | 0% | cephalosporin, monobactam and carbapenems |  |
| PDC-430 | 0% | cephalosporin, monobactam and carbapenems |  |
| PDC-45 | 0% | cephalosporin, monobactam and carbapenems |  |
| PDC-5 | 14% | cephalosporin, monobactam and carbapenems |  |
| PDC-60 | 1% | cephalosporin, monobactam and carbapenems |  |
| PDC-72 | 0% | cephalosporin, monobactam and carbapenems |  |
| PDC-80 | < 1% | cephalosporin, monobactam and carbapenems |  |
| PDC-95 | unknown | cephalosporin, monobactam and carbapenems |  |
| PDC-95 | 0% | cephalosporin, monobactam and carbapenems |  |

| **Table S3. Accumulated mutations of each patient's longitudinal isolate compared to the ancestral (first isolate) isolate.** | | | |  |
| --- | --- | --- | --- | --- |
|  |  |  |  |  |
|  | Isolation date | Isolate | Accumulated variants |  |
| Patient 1 | 02/27/14 | A703 | 27 |  |
|  | 12/12/14 | A705 | 32 |  |
|  | 04/16/15 | A701 | 30 |  |
|  | 12/10/15 | A710 | 36 |  |
|  | 04/19/16 | A734 | 36 |  |
|  | 09/02/16 | A748 | 37 |  |
|  | 04/24/17 | A768 | 41 |  |
|  | 11/22/17 | A780 | 46 |  |
|  | 10/12/18 | AG774 | 46 |  |
|  | 10/12/18 | AP774 | 44 |  |
|  | 07/02/21 | A2152 | 75 |  |
|  | 11/03/21 | A2155 | 74 |  |
|  | 02/09/22 | A2158 | 82 |  |
|  | 06/28/22 | AG2160 | 53 |  |
|  | 06/28/22 | AP2160 | 52 |  |
|  | 01/09/23 | A2162 | 56 |  |
| Patient 2 | 04/28/14 | A693 | 0 |  |
|  | 04/23/15 | A700 | 99 |  |
|  | 12/02/14 | A709 | 15 |  |
|  | 12/11/15 | A725 | 23 |  |
|  | 12/16/16 | A743 | 127 |  |
|  | 06/30/17 | A766 | 528 |  |
| Patient 3 | 12/05/14 | A708 | 0 |  |
|  | 09/13/16 | A747 | 38 |  |
|  | 07/12/17 | A765 | 40 |  |
|  | 04/19/16 | AM723 | 5 |  |
|  | 04/19/16 | AP723 | 35 |  |

| **Table S4. Number of genes and lenght of the whole genome, mobilome elements, and accessory genome of the included isolates.** | | | | | | | | | | | | | | |
| --- | --- | --- | --- | --- | --- | --- | --- | --- | --- | --- | --- | --- | --- | --- |
| Isolate | Isolation date | Patient | Total Genome Length | Num Genes (prodigal) | Phage sequences lenght | Phage sequences | Phage genes (prodigal) | MGE sequences lenght | MGE sequences | MGE genes | Core genes | Accessory genes | Mobilome genes | Accessory genes - mobilome genes |
| A697 | 01/04/2013 | 1 | 6857866 | 6410 | 98793 | 4 | 138 | 171121 | 11 | 195 | 5258 | 1152 | 333 | 819 |
| A703 | 27/02/2014 | 1 | 6858411 | 6415 | 94834 | 4 | 131 | 171121 | 11 | 196 | 5258 | 1157 | 327 | 830 |
| A705 | 12/12/2014 | 1 | 6858354 | 6422 | 108426 | 4 | 151 | 171120 | 11 | 196 | 5258 | 1164 | 347 | 817 |
| A701 | 16/04/2015 | 1 | 6780593 | 6344 | 106365 | 4 | 149 | 171120 | 11 | 195 | 5258 | 1086 | 344 | 742 |
| A710 | 10/12/2015 | 1 | 6858403 | 6422 | 102406 | 4 | 142 | 171120 | 11 | 196 | 5258 | 1164 | 338 | 826 |
| A734 | 19/04/2016 | 1 | 6858388 | 6418 | 94834 | 4 | 131 | 171121 | 11 | 196 | 5258 | 1160 | 327 | 833 |
| A748 | 02/09/2016 | 1 | 6858338 | 6418 | 102406 | 4 | 142 | 171063 | 11 | 196 | 5258 | 1160 | 338 | 822 |
| A768 | 24/04/2017 | 1 | 6857521 | 6420 | 104467 | 4 | 144 | 171121 | 11 | 196 | 5258 | 1162 | 340 | 822 |
| A780 | 22/11/2017 | 1 | 6857499 | 6416 | 102406 | 4 | 142 | 171121 | 11 | 195 | 5258 | 1158 | 337 | 821 |
| A778 | 02/03/2018 | 1 | 6363977 | 5922 | 35194 | 2 | 46 | 52073 | 4 | 53 | 5258 | 664 | 99 | 565 |
| AG774 | 12/10/2018 | 1 | 6856958 | 6420 | 94834 | 4 | 131 | 171121 | 11 | 196 | 5258 | 1162 | 327 | 835 |
| AP774 | 12/10/2018 | 1 | 6856749 | 6419 | 98793 | 4 | 138 | 171121 | 11 | 195 | 5258 | 1161 | 333 | 828 |
| A771 | 11/11/2020 | 1 | 6420009 | 6000 | 70025 | 3 | 94 | 54826 | 6 | 56 | 5258 | 742 | 150 | 592 |
| A2152 | 02/07/2021 | 1 | 6856574 | 6415 | 98793 | 4 | 138 | 171120 | 11 | 195 | 5258 | 1157 | 333 | 824 |
| A2155 | 03/11/2021 | 1 | 6856487 | 6413 | 104467 | 4 | 144 | 171145 | 11 | 195 | 5258 | 1155 | 339 | 816 |
| A2158 | 09/02/2022 | 1 | 6856697 | 6416 | 108426 | 4 | 151 | 171121 | 11 | 196 | 5258 | 1158 | 347 | 811 |
| AG2160 | 28/06/2022 | 1 | 6857474 | 6417 | 106365 | 4 | 149 | 171120 | 11 | 195 | 5258 | 1159 | 344 | 815 |
| AP2160 | 28/06/2022 | 1 | 6856824 | 6421 | 100854 | 4 | 140 | 171120 | 11 | 195 | 5258 | 1163 | 335 | 828 |
| A2162 | 09/01/2023 | 1 | 6770307 | 6343 | 102406 | 4 | 142 | 171120 | 11 | 195 | 5258 | 1085 | 337 | 748 |
| A693 | 28/04/2014 | 2 | 6364344 | 5912 | 77698 | 2 | 107 | 57692 | 8 | 59 | 5258 | 654 | 166 | 488 |
| A709 | 02/12/2014 | 2 | 6363669 | 5909 | 91461 | 2 | 127 | 57692 | 8 | 58 | 5258 | 651 | 185 | 466 |
| A700 | 23/04/2015 | 2 | 6363063 | 5913 | 91462 | 2 | 126 | 57692 | 8 | 58 | 5258 | 655 | 184 | 471 |
| A725 | 11/12/2015 | 2 | 6364656 | 5917 | 77993 | 2 | 107 | 57692 | 8 | 59 | 5258 | 659 | 166 | 493 |
| A743 | 16/12/2016 | 2 | 6363234 | 5917 | 77698 | 2 | 107 | 57693 | 8 | 59 | 5258 | 659 | 166 | 493 |
| A766 | 30/06/2017 | 2 | 6362657 | 5915 | 77698 | 2 | 107 | 57692 | 8 | 59 | 5258 | 657 | 166 | 491 |
| A708 | 05/12/2014 | 3 | 6331259 | 5852 | 17420 | 1 | 25 | 55966 | 8 | 62 | 5258 | 594 | 87 | 507 |
| AM723 | 19/04/2016 | 3 | 6330595 | 5852 | 17420 | 1 | 25 | 55966 | 8 | 61 | 5258 | 594 | 86 | 508 |
| AP723 | 19/04/2016 | 3 | 6325678 | 5843 | 17420 | 1 | 25 | 55965 | 8 | 62 | 5258 | 585 | 87 | 498 |
| A747 | 13/09/2016 | 3 | 6324596 | 5844 | 17420 | 1 | 25 | 55966 | 8 | 62 | 5258 | 586 | 87 | 499 |
| A765 | 12/07/2017 | 3 | 6324442 | 5840 | 17420 | 1 | 25 | 55966 | 8 | 61 | 5258 | 582 | 86 | 496 |
| B972 | 11/07/2019 | 4 | 6793708 | 6405 | 125664 | 4 | 192 | 57265 | 7 | 59 | 5258 | 1147 | 251 | 896 |
| A961 | 08/03/2020 | 4 | 6784204 | 6411 | 125664 | 4 | 191 | 57264 | 7 | 59 | 5258 | 1153 | 250 | 903 |
| B699 | 23/01/2012 | 5 | 5832038 | 5429 | 20226 | 1 | 27 | 49577 | 2 | 50 | 5258 | 171 | 77 | 94 |
| A773 | 06/03/2019 | 6 | 6357696 | 5875 | 111163 | 3 | 149 | 49730 | 2 | 51 | 5258 | 617 | 200 | 417 |
| A776 | 18/05/2018 | 7 | 6300526 | 5859 | 74696 | 4 | 105 | 51097 | 3 | 53 | 5258 | 601 | 158 | 443 |
| A949 | 02/04/2018 | 8 | 6303928 | 5787 | 27998 | 2 | 42 | 53328 | 5 | 55 | 5258 | 529 | 97 | 432 |
| A942 | 19/05/2018 | 9 | 6424855 | 5981 | 76336 | 3 | 104 | 56072 | 6 | 57 | 5258 | 723 | 161 | 562 |
| A928 | 06/07/2018 | 10 | 6340274 | 5872 | 45775 | 3 | 64 | 54471 | 5 | 57 | 5258 | 614 | 121 | 493 |
| A931 | 30/07/2019 | 11 | 6348313 | 5892 | 60798 | 2 | 85 | 49994 | 2 | 50 | 5258 | 634 | 135 | 499 |

| **Table S5. Phage sequences identified by the PHASTEST software and BLASTN results when comparing R- and F-type pyocins sequences against the correspoding phage sequence.** | | | | | |  |
| --- | --- | --- | --- | --- | --- | --- |
|  |  |  |  |  |  |  |
| Isolate | Patient | Isolation date | Most similar phage | Completeness | BLASTN Pyocin hit |  |
| A697 | Patient 1 | 4/1/2013 | PHAGE_Pseudo_YMC11/02/R656_NC_028657 | questionable | F-pyocin |  |
| A697 | Patient 1 | 4/1/2013 | PHAGE_Pseudo_JBD67_NC_042135 | questionable | N/A |  |
| A697 | Patient 1 | 4/1/2013 | PHAGE_Pseudo_Pf1_NC_001331 | intact | N/A |  |
| A697 | Patient 1 | 4/1/2013 | PHAGE_Pseudo_phi297_NC_016762 | questionable | N/A |  |
| A703 | Patient 1 | 2/27/2014 | PHAGE_Pseudo_YMC11/02/R656_NC_028657 | questionable | F-pyocin |  |
| A703 | Patient 1 | 2/27/2014 | PHAGE_Pseudo_JBD25_NC_027992 | questionable | N/A |  |
| A703 | Patient 1 | 2/27/2014 | PHAGE_Pseudo_Pf1_NC_001331 | intact | N/A |  |
| A703 | Patient 1 | 2/27/2014 | PHAGE_Pseudo_phi297_NC_016762 | questionable | N/A |  |
| A705 | Patient 1 | 12/12/2014 | PHAGE_Pseudo_YMC11/02/R656_NC_028657 | questionable | F-pyocin |  |
| A705 | Patient 1 | 12/12/2014 | PHAGE_Pseudo_JBD25_NC_027992 | questionable | N/A |  |
| A705 | Patient 1 | 12/12/2014 | PHAGE_Pseudo_Pf1_NC_001331 | intact | N/A |  |
| A705 | Patient 1 | 12/12/2014 | PHAGE_Pseudo_phi297_NC_016762 | questionable | N/A |  |
| A701 | Patient 1 | 4/16/2015 | PHAGE_Pseudo_YMC11/02/R656_NC_028657 | questionable | F-pyocin |  |
| A701 | Patient 1 | 4/16/2015 | PHAGE_Pseudo_JBD67_NC_042135 | questionable | N/A |  |
| A701 | Patient 1 | 4/16/2015 | PHAGE_Pseudo_Pf1_NC_001331 | intact | N/A |  |
| A701 | Patient 1 | 4/16/2015 | PHAGE_Pseudo_phi297_NC_016762 | questionable | N/A |  |
| A710 | Patient 1 | 12/10/2015 | PHAGE_Pseudo_YMC11/02/R656_NC_028657 | questionable | F-pyocin |  |
| A710 | Patient 1 | 12/10/2015 | PHAGE_Pseudo_JBD25_NC_027992 | questionable | N/A |  |
| A710 | Patient 1 | 12/10/2015 | PHAGE_Pseudo_Pf1_NC_001331 | intact | N/A |  |
| A710 | Patient 1 | 12/10/2015 | PHAGE_Pseudo_phi297_NC_016762 | questionable | N/A |  |
| A734 | Patient 1 | 4/19/2016 | PHAGE_Pseudo_YMC11/02/R656_NC_028657 | questionable | F-pyocin |  |
| A734 | Patient 1 | 4/19/2016 | PHAGE_Pseudo_JBD67_NC_042135 | questionable | N/A |  |
| A734 | Patient 1 | 4/19/2016 | PHAGE_Pseudo_Pf1_NC_001331 | intact | N/A |  |
| A734 | Patient 1 | 4/19/2016 | PHAGE_Pseudo_phi297_NC_016762 | questionable | N/A |  |
| A748 | Patient 1 | 9/2/2016 | PHAGE_Pseudo_YMC11/02/R656_NC_028657 | questionable | F-pyocin |  |
| A748 | Patient 1 | 9/2/2016 | PHAGE_Pseudo_JBD25_NC_027992 | questionable | N/A |  |
| A748 | Patient 1 | 9/2/2016 | PHAGE_Pseudo_Pf1_NC_001331 | intact | N/A |  |
| A748 | Patient 1 | 9/2/2016 | PHAGE_Pseudo_phi297_NC_016762 | questionable | N/A |  |
| A768 | Patient 1 | 4/24/2017 | PHAGE_Pseudo_YMC11/02/R656_NC_028657 | incomplete | F-pyocin |  |
| A768 | Patient 1 | 4/24/2017 | PHAGE_Pseudo_JBD67_NC_042135 | questionable | N/A |  |
| A768 | Patient 1 | 4/24/2017 | PHAGE_Pseudo_Pf1_NC_001331 | intact | N/A |  |
| A780 | Patient 1 | 11/22/2017 | PHAGE_Pseudo_YMC11/02/R656_NC_028657 | questionable | F-pyocin |  |
| A780 | Patient 1 | 11/22/2017 | PHAGE_Pseudo_JBD25_NC_027992 | questionable | N/A |  |
| A780 | Patient 1 | 11/22/2017 | PHAGE_Pseudo_Pf1_NC_001331 | intact | N/A |  |
| A780 | Patient 1 | 11/22/2017 | PHAGE_Pseudo_phi297_NC_016762 | questionable | N/A |  |
| A778 | Patient 1 | 3/2/2018 | PHAGE_Pseudo_YMC11/02/R656_NC_028657 | questionable | F-pyocin |  |
| A778 | Patient 1 | 3/2/2018 | PHAGE_Pseudo_Pf1_NC_001331 | questionable | N/A |  |
| AG774 | Patient 1 | 10/12/2018 | PHAGE_Pseudo_YMC11/02/R656_NC_028657 | questionable | F-pyocin |  |
| AG774 | Patient 1 | 10/12/2018 | PHAGE_Pseudo_JBD67_NC_042135 | questionable | N/A |  |
| AG774 | Patient 1 | 10/12/2018 | PHAGE_Pseudo_Pf1_NC_001331 | intact | N/A |  |
| AG774 | Patient 1 | 10/12/2018 | PHAGE_Pseudo_phi297_NC_016762 | questionable | N/A |  |
| AP774 | Patient 1 | 10/12/2018 | PHAGE_Pseudo_YMC11/02/R656_NC_028657 | questionable | F-pyocin |  |
| AP774 | Patient 1 | 10/12/2018 | PHAGE_Pseudo_JBD25_NC_027992 | questionable | N/A |  |
| AP774 | Patient 1 | 10/12/2018 | PHAGE_Pseudo_Pf1_NC_001331 | intact | N/A |  |
| AP774 | Patient 1 | 10/12/2018 | PHAGE_Pseudo_phi297_NC_016762 | questionable | N/A |  |
| A771 | Patient 1 | 11/11/2020 | PHAGE_Pseudo_phiCTX_NC_003278 | incomplete | R-pyocin |  |
| A771 | Patient 1 | 11/11/2020 | PHAGE_Stenot_S1_NC_011589 | incomplete | N/A |  |
| A2152 | Patient 1 | 7/2/2021 | PHAGE_Pseudo_YMC11/02/R656_NC_028657 | questionable | F-pyocin |  |
| A2152 | Patient 1 | 7/2/2021 | PHAGE_Pseudo_JBD67_NC_042135 | questionable | N/A |  |
| A2152 | Patient 1 | 7/2/2021 | PHAGE_Pseudo_Pf1_NC_001331 | intact | N/A |  |
| A2152 | Patient 1 | 7/2/2021 | PHAGE_Pseudo_phi297_NC_016762 | questionable | N/A |  |
| A2155 | Patient 1 | 11/3/2021 | PHAGE_Pseudo_YMC11/02/R656_NC_028657 | questionable | F-pyocin |  |
| A2155 | Patient 1 | 11/3/2021 | PHAGE_Pseudo_JBD25_NC_027992 | questionable | N/A |  |
| A2155 | Patient 1 | 11/3/2021 | PHAGE_Pseudo_Pf1_NC_001331 | intact | N/A |  |
| A2155 | Patient 1 | 11/3/2021 | PHAGE_Pseudo_phi297_NC_016762 | questionable | N/A |  |
| A2158 | Patient 1 | 2/9/2022 | PHAGE_Pseudo_YMC11/02/R656_NC_028657 | questionable | F-pyocin |  |
| A2158 | Patient 1 | 2/9/2022 | PHAGE_Pseudo_JBD25_NC_027992 | questionable | N/A |  |
| A2158 | Patient 1 | 2/9/2022 | PHAGE_Pseudo_Pf1_NC_001331 | intact | N/A |  |
| A2158 | Patient 1 | 2/9/2022 | PHAGE_Pseudo_phi297_NC_016762 | questionable | N/A |  |
| AG2160 | Patient 1 | 6/28/2022 | PHAGE_Pseudo_YMC11/02/R656_NC_028657 | questionable | F-pyocin |  |
| AG2160 | Patient 1 | 6/28/2022 | PHAGE_Pseudo_JBD25_NC_027992 | questionable | N/A |  |
| AG2160 | Patient 1 | 6/28/2022 | PHAGE_Pseudo_Pf1_NC_001331 | intact | N/A |  |
| AG2160 | Patient 1 | 6/28/2022 | PHAGE_Pseudo_phi297_NC_016762 | questionable | N/A |  |
| AP2160 | Patient 1 | 6/28/2022 | PHAGE_Pseudo_YMC11/02/R656_NC_028657 | questionable | F-pyocin |  |
| AP2160 | Patient 1 | 6/28/2022 | PHAGE_Pseudo_JBD67_NC_042135 | questionable | N/A |  |
| AP2160 | Patient 1 | 6/28/2022 | PHAGE_Pseudo_Pf1_NC_001331 | intact | N/A |  |
| AP2160 | Patient 1 | 6/28/2022 | PHAGE_Pseudo_phi297_NC_016762 | questionable | N/A |  |
| A2162 | Patient 1 | 1/9/2023 | PHAGE_Pseudo_YMC11/02/R656_NC_028657 | questionable | F-pyocin |  |
| A2162 | Patient 1 | 1/9/2023 | PHAGE_Pseudo_JBD25_NC_027992 | questionable | N/A |  |
| A2162 | Patient 1 | 1/9/2023 | PHAGE_Pseudo_Pf1_NC_001331 | intact | N/A |  |
| A2162 | Patient 1 | 1/9/2023 | PHAGE_Pseudo_phi297_NC_016762 | questionable | N/A |  |
| A693 | Patient 2 | 4/28/2014 | PHAGE_Pseudo_YMC11/02/R656_NC_028657 | intact | Both |  |
| A693 | Patient 2 | 4/28/2014 | PHAGE_Pseudo_YMC11/07/P54_PAE_BP_NC_030909 | intact | F-pyocin |  |
| A709 | Patient 2 | 12/2/2014 | PHAGE_Pseudo_YMC11/02/R656_NC_028657 | intact | Both |  |
| A709 | Patient 2 | 12/2/2014 | PHAGE_Pseudo_YMC11/02/R656_NC_028657 | intact | F-pyocin |  |
| A700 | Patient 2 | 4/23/2015 | PHAGE_Pseudo_YMC11/02/R656_NC_028657 | intact | Both |  |
| A700 | Patient 2 | 4/23/2015 | PHAGE_Pseudo_YMC11/02/R656_NC_028657 | intact | F-pyocin |  |
| A725 | Patient 2 | 12/11/2015 | PHAGE_Pseudo_YMC11/02/R656_NC_028657 | intact | Both |  |
| A725 | Patient 2 | 12/11/2015 | PHAGE_Pseudo_YMC11/02/R656_NC_028657 | intact | F-pyocin |  |
| A743 | Patient 2 | 12/16/2016 | PHAGE_Pseudo_YMC11/02/R656_NC_028657 | intact | Both |  |
| A743 | Patient 2 | 12/16/2016 | PHAGE_Pseudo_YMC11/07/P54_PAE_BP_NC_030909 | intact | F-pyocin |  |
| A766 | Patient 2 | 6/30/2017 | PHAGE_Pseudo_YMC11/02/R656_NC_028657 | intact | Both |  |
| A766 | Patient 2 | 6/30/2017 | PHAGE_Pseudo_YMC11/07/P54_PAE_BP_NC_030909 | intact | F-pyocin |  |
| A708 | Patient 3 | 12/5/2014 | PHAGE_Pseudo_Dobby_NC_048109 | intact | R-pyocin |  |
| AM723 | Patient 3 | 4/19/2016 | PHAGE_Entero_Arya_NC_031048 | intact | R-pyocin |  |
| AP723 | Patient 3 | 4/19/2016 | PHAGE_Entero_Arya_NC_031048 | intact | R-pyocin |  |
| A747 | Patient 3 | 9/13/2016 | PHAGE_Pseudo_Dobby_NC_048109 | intact | R-pyocin |  |
| A765 | Patient 3 | 7/12/2017 | PHAGE_Pseudo_phiCTX_NC_003278 | intact | R-pyocin |  |
| B972 | Patient 4 | 7/11/2019 | PHAGE_Pseudo_YMC11/02/R656_NC_028657 | intact | Both |  |
| B972 | Patient 4 | 7/11/2019 | PHAGE_Pseudo_F10_NC_007805 | intact | N/A |  |
| B972 | Patient 4 | 7/11/2019 | PHAGE_Pseudo_JBD24_NC_020203 | intact | N/A |  |
| B972 | Patient 4 | 7/11/2019 | PHAGE_Pseudo_PMG1_NC_016765 | intact | N/A |  |
| A961 | Patient 4 | 3/8/2020 | PHAGE_Pseudo_YMC11/02/R656_NC_028657 | intact | Both |  |
| A961 | Patient 4 | 3/8/2020 | PHAGE_Pseudo_F10_NC_007805 | intact | N/A |  |
| A961 | Patient 4 | 3/8/2020 | PHAGE_Pseudo_JBD24_NC_020203 | intact | N/A |  |
| A961 | Patient 4 | 3/8/2020 | PHAGE_Pseudo_PMG1_NC_016765 | intact | N/A |  |
| B699 | Patient 5 | 1/23/2012 | PHAGE_Pseudo_YMC11/02/R656_NC_028657 | intact | F-pyocin |  |
| A773 | Patient 6 | 43530 | PHAGE_Pseudo_YMC11/02/R656_NC_028657 | intact | Both |  |
| A773 | Patient 6 | 3/6/2019 | PHAGE_Pseudo_B3_NC_006548 | intact | N/A |  |
| A773 | Patient 6 | 3/6/2019 | PHAGE_Pseudo_PMG1_NC_016765 | intact | N/A |  |
| A776 | Patient 7 | 5/18/2018 | PHAGE_Pseudo_YMC11/02/R656_NC_028657 | questionable | F-pyocin |  |
| A776 | Patient 7 | 5/18/2018 | PHAGE_Pseudo_Dobby_NC_048109 | intact | R-pyocin |  |
| A776 | Patient 7 | 5/18/2018 | PHAGE_Pseudo_Pf1_NC_001331 | questionable | N/A |  |
| A776 | Patient 7 | 5/18/2018 | PHAGE_Pseudo_phi2_NC_030931 | intact | N/A |  |
| A928 | Patient 8 | 7/6/2018 | PHAGE_Pseudo_YMC11/02/R656_NC_028657 | intact | F-pyocin |  |
| A928 | Patient 8 | 7/6/2018 | PHAGE_Pseudo_Pf1_NC_001331 | questionable | N/A |  |
| A928 | Patient 8 | 7/6/2018 | PHAGE_Pseudo_Pf1_NC_001331 | intact | N/A |  |
| A931 | Patient 9 | 7/30/2019 | PHAGE_Pseudo_phiCTX_NC_003278 | intact | R-pyocin |  |
| A931 | Patient 9 | 7/30/2019 | PHAGE_Pseudo_phi297_NC_016762 | intact | N/A |  |
| A942 | Patient 10 | 5/19/2018 | PHAGE_Pseudo_phi297_NC_016762 | intact | F-pyocin |  |
| A942 | Patient 10 | 5/19/2018 | PHAGE_Pseudo_YMC11/02/R656_NC_028657 | questionable | F-pyocin |  |
| A942 | Patient 10 | 5/19/2018 | PHAGE_Pseudo_Pf1_NC_001331 | intact | N/A |  |
| A949 | Patient 11 | 4/2/2018 | PHAGE_Pseudo_Dobby_NC_048109 | intact | R-pyocin |  |
| A949 | Patient 11 | 4/2/2018 | PHAGE_Pseudo_Pf1_NC_001331 | intact | N/A |  |

| **Table S6. Number of mobilome elements identified for each individual sequence type (ST).** | | | |  |
| --- | --- | --- | --- | --- |
|  |  |  |  |  |
| Patient | ST | Phage sequences | MGE sequences |  |
| 1 | 829 | 4 | 11 |  |
|  | 934 | 2 | 4 |  |
|  | 1800 | 3 | 6 |  |
| 2 | 244 | 2 | 8 |  |
| 3 | 2234 | 1 | 8 |  |
| 4 | 381 | 4 | 7 |  |
| 5 | 4052 | 1 | 2 |  |
| 6 | 2604 | 3 | 2 |  |
| 7 | 274 | 4 | 3 |  |
| 8 | 155 | 2 | 5 |  |
| 9 | 254 | 3 | 6 |  |
| 10 | 3426 | 3 | 5 |  |
| 11 | Novel | 2 | 2 |  |

| **Table S7. Mobile genetic elements identified, genome count, and associated functions according to the corresponding database (IS: insertion sequence; ICE: integrative conjugative element; Tn: transposon).** | | | | | |  |
| --- | --- | --- | --- | --- | --- | --- |
|  |  |  |  |  |  |  |
| MGE | Count | Type | Database | Accession ID | Associated functions |  |
| IS222 | 16 | IS | ISFinder | AFXK01000001 | Transposase |  |
| ISBcen18 | 2 | IS | ISFinder | N/A | Transposase |  |
| ISPa1 | 13 | IS | ISFinder | M27175 | Transposase |  |
| ISPa11 | 17 | IS | ISFinder | AE004697 | Transposase |  |
| ISPa123 | 5 | IS | ISFinder | CP041774 | Transposase |  |
| ISPa124 | 5 | IS | ISFinder | CP041774.1 | Transposase |  |
| ISPa127 | 17 | IS | ISFinder | N/A | Transposase |  |
| ISPa128 | 2 | IS | ISFinder | CP054623.1 | GNAT family N-acetyl transferase |  |
| ISPa131 | 1 | IS | ISFinder | CP041354 | Transposase |  |
| ISPa2 | 6 | IS | ISFinder | M27186 | Exotoxin A |  |
| ISPa22 | 6 | IS | ISFinder | NC_002516 | Transposase |  |
| ISPa26 | 2 | IS | ISFinder | EU000222 | Transposase |  |
| ISPa32 | 39 | IS | ISFinder | NC_002516 | Transposase |  |
| ISPa37 | 26 | IS | ISFinder | AFXK01000001 | Transposase |  |
| ISPa4 | 1 | IS | ISFinder | U16785 | Exotoxin A |  |
| ISPa41 | 17 | IS | ISFinder | N/A | Transposase |  |
| ISPa5 | 5 | IS | ISFinder | [U16785](http://www.ncbi.nlm.nih.gov/nuccore/U16785) | Exotoxin A |  |
| ISPa6 | 27 | IS | ISFinder | U16784 | N/A |  |
| ISPa97 | 1 | IS | ISFinder | EU595745 | Transposase |  |
| ISPen2 | 17 | IS | ISFinder | NC_008027 | Transposase |  |
| ISPpu17 | 2 | IS | ISFinder | N/A | Transposase |  |
| ISPsy29 | 2 | IS | ISFinder | NC_007274 | Transposase |  |
| PAGI-5 | 17 | ICE | ICEberg 3.0 | ICEO_0000117 | Mercury resistance |  |
| Tn4371 | 39 | ICE | ICEberg 3.0 | ICEO_0000097 | Biphenly degradation |  |
| Tn4656 | 17 | Tn | The transposon registry | AB062597 | Toluene degradation |  |
| Tn4661 | 17 | Tn | The transposon registry | AB375440 | Transposase |  |

| **Table S8. Specific growth rate of the included isolates. Experiments were performed by quadruplicate on at least two different occassions.** | | | |  |
| --- | --- | --- | --- | --- |
|  |  |  |  |  |
| Isolate | Patient | Isolation date | Specific growth rate |  |
| A697 | 1 | 01/04/2013 | 0.078 |  |
| A703 | 1 | 27/02/2014 | 0.087 |  |
| A705 | 1 | 12/12/2014 | 0.057 |  |
| A701 | 1 | 16/04/2015 | 0.091 |  |
| A710 | 1 | 10/12/2015 | 0.066 |  |
| A734 | 1 | 19/04/2016 | 0.077 |  |
| A748 | 1 | 02/09/2016 | 0.086 |  |
| A768 | 1 | 24/04/2017 | 0.075 |  |
| A780 | 1 | 22/11/2017 | 0.064 |  |
| A778 | 1 | 02/03/2018 | 0.100 |  |
| AG774 | 1 | 12/10/2018 | 0.078 |  |
| AP774 | 1 | 12/10/2018 | 0.073 |  |
| A771 | 1 | 11/11/2020 | 0.079 |  |
| A2152 | 1 | 02/07/2021 | 0.041 |  |
| A2155 | 1 | 03/11/2021 | 0.051 |  |
| A2158 | 1 | 09/02/2022 | 0.053 |  |
| AG2160 | 1 | 28/06/2022 | 0.038 |  |
| AP2160 | 1 | 28/06/2022 | 0.038 |  |
| A2162 | 1 | 09/01/2023 | 0.045 |  |
| A693 | 2 | 28/04/2014 | 0.078 |  |
| A709 | 2 | 02/12/2014 | 0.084 |  |
| A700 | 2 | 23/04/2015 | 0.031 |  |
| A725 | 2 | 11/12/2015 | 0.085 |  |
| A743 | 2 | 16/12/2016 | 0.041 |  |
| A766 | 2 | 30/06/2017 | 0.039 |  |
| A708 | 3 | 05/12/2014 | 0.062 |  |
| AM723 | 3 | 19/04/2016 | 0.069 |  |
| AP723 | 3 | 19/04/2016 | 0.062 |  |
| A747 | 3 | 13/09/2016 | 0.058 |  |
| A765 | 3 | 12/07/2017 | 0.053 |  |
| B972 | 4 | 11/07/2019 | 0.034 |  |
| A961 | 4 | 08/03/2020 | 0.039 |  |
| B699 | 5 | 23/01/2012 | 0.053 |  |
| A773 | 6 | 06/03/2019 | 0.064 |  |
| A776 | 7 | 18/05/2018 | 0.105 |  |
| A949 | 8 | 02/04/2018 | 0.049 |  |
| A942 | 9 | 19/05/2018 | 0.062 |  |
| A928 | 10 | 06/07/2018 | 0.071 |  |
| A931 | 11 | 30/07/2019 | 0.081 |  |
